# Erratum to “Harnessing Nanotechnology for Gout Therapy: Colchicine-Loaded Nanoparticles Regulate Macrophage Polarization and Reduce Inflammation”

**DOI:** 10.34133/bmr.0141

**Published:** 2025-03-05

**Authors:** Yu Chen, Lanqing Zhao, Jinwei Li, Hongxi Li, Ning Zhang

**Affiliations:** ^1^ Department of The Fourth Otolaryngology Head and Neck Surgery, Shengjing Hospital of China Medical University, Shenyang 110000, China.; ^2^ Department of Sleep Medicine Center, The Shengjing Affiliated Hospital, China Medical University, Shenyang 110000, Liaoning, China.; ^3^ Department of Neurology/Stroke Center, the First Affiliated Hospital of China Medical University, China Medical University, Shenyang 110000, Liaoning, China.; ^4^ Department of Pain Management, Shengjing Hospital of China Medical University, Shenyang 110000, China.; ^5^ Department of Rheumatology and Immunology, Shengjing Hospital Affiliated to China Medical University, Shenyang 110000, China.

In the Research Article “Harnessing Nanotechnology for Gout Therapy: Colchicine-Loaded Nanoparticles Regulate Macrophage Polarization and Reduce Inflammation,” a change is needed in the author list [1]. The original author list has been changed to accurately reflect the contributions of each author.

The original author list was Ning Zhang^†^, Lanqing Zhao^†^, Jinwei Li^†^, Hongxi Li^†^, and Yu Chen^*^

The corrected order for authorship is Yu Chen, Lanqing Zhao^†^, Jinwei Li^†^, Hongxi Li^†^, and Ning Zhang^†*^

Given the extensive guidance, supervision, and contributions provided by Prof. Zhang Ning during the research and writing process, the authors believe that it is more appropriate and consistent with academic standards for Zhang Ning to be recognized as the *corresponding* author and Yu Chen to be listed as the *first* author.

This change does not affect the findings of this work, and relevant correspondence should be directed to Prof. Zhang Ning (zhangning@sj-hospital.org).
