# Supplementary material for: Harnessing Nanotechnology for Gout Therapy: Colchicine-Loaded Nanoparticles Regulate Macrophage Polarization and Reduce Inflammation
Source: Biomater Res. 2024 Dec 11;28:0089. doi: 10.34133/bmr.0089 (PMC11632155; doi:10.34133/bmr.0089)
Supplement: 20241211-1 [file bmr.0089.v1.pdf]

Biomaterials Research
A SCIENCE PARTNER JOURNAL

RESEARCH ARTICLE

Harnessing Nanotechnology for Gout Therapy: Colchicine-Loaded Nanoparticles Regulate Macrophage Polarization and Reduce Inflammation

Ning Zhang[1†], Lanqing Zhao[2†], Jinwei Li[3†], Hongxi Li[4†], and Yu Chen[5*]

[1]Department of Rheumatology and Immunology, Shengjing Hospital Affiliated to China Medical University, Shenyang 110000, China. [2]Department of Sleep Medicine Center, The Shengjing Affiliated Hospital, China Medical University, Shenyang 110000, Liaoning, China. [3]Department of Neurology/Stroke Center, the First Affiliated Hospital ofChina Medical University, China Medical University, Shenyang 110000, Liaoning, China. [4]Department of Pain Management, Shengjing Hospital of China Medical University, Shenyang 110000, China. [5]Department of The Fourth Otolaryngology Head and Neck Surgery, Shengjing Hospital of China Medical University, Shenyang 110000, China.

*Address correspondence to: chenyu870720@163.com
†These authors contributed equally to this work.

Citation: Zhang N, Zhao L, Li J, Li H, Chen Y. Harnessing Nanotechnology for Gout Therapy: Colchicine-Loaded Nanoparticles Regulate Macrophage Polarization and Reduce Inflammation. *Biomater. Res.* 2024;28:Article 0089. https://doi.org/10.34133/bmr.0089

Submitted 25 March 2024
Revised 13 September 2024
Accepted 24 September 2024
Published 11 December 2024

Copyright © 2024 Ning Zhang et al. Exclusive licensee Korean Society for Biomaterials, Republic of Korea. No claim to original U.S. Government Works. Distributed under a Creative Commons Attribution License (CC BY 4.0).

Gout is a disease caused by hyperuricemia, characterized by inflammation reactions triggered by macrophage polarization. Colchicine is a commonly used drug for gout treatment, but its mechanism of action remains unclear. The aim of this study was to investigate the regulatory effect of colchicine on macrophage polarization to enhance the therapeutic effectiveness against gout inflammation. To accomplish this, a mouse model was established, and peripheral blood mononuclear cell samples were collected. Single-cell RNA sequencing was employed to reveal cellular heterogeneity and identify key genes. Molecular docking and experimental validation were performed to confirm the binding between the key genes and colchicine. Lentiviral intervention and biochemical indicator detection were conducted to assess the impact of key genes on gout mice. Additionally, the therapeutic effect of colchicine incorporated into neutrophil membrane-coated nanoparticles was investigated. The study found that macrophage polarization plays a critical role in gout, and AHNAK was identified as the key gene through which colchicine affects macrophage polarization. Lentiviral intervention to decrease AHNAK expression was shown to alleviate joint swelling in gout mice and regulate macrophage polarization. Colchicine encapsulated in R4F peptide-modified neutrophil membrane-coated Pluronic F127 nanoparticle (R4F-NM@F127) nanocarriers inhibited M1 macrophage polarization, induced M2 macrophage polarization, alleviated gout, and minimized toxicity to normal tissues. Colchicine suppressed M1 macrophage polarization and induced M2 macrophage polarization by binding to AHNAK protein, thereby alleviating gout. Colchicine incorporated into R4F-NM@F127 nanocarriers can serve as a targeted therapeutic drug to regulate macrophage polarization, alleviate gout, and reduce toxicity to normal tissues.

Introduction

Gout is a metabolic disorder primarily characterized by elevated levels of uric acid in the blood [1]. The deposition of urate crystals in the joints and surrounding tissues contributes to its pathogenesis [2]. Common symptoms of gout include acute joint inflammation and uric acid kidney stones, significantly impacting patients' quality of life and health [3,4]. Current treatment approaches for gout typically involve nonsteroidal anti-inflammatory drugs, steroids, and urate-lowering agents. However, traditional therapies are associated with various limitations, such as drug side effects and inconsistent efficacy [5,6].

Macrophages play a crucial role in gout inflammation [7,8]. As important components of the immune system, macrophages contribute to both innate and adaptive immune responses and play a critical role in maintaining tissue homeostasis [9,10]. Macrophage polarization is an essential mechanism for macrophage functional regulation. Depending on the environmental stimuli, macrophages can polarize into pro-inflammatory M1 or anti-inflammatory M2 phenotypes [11,12]. Macrophage polarization plays an important role in gout inflammation [7,13].

Colchicine is a widely used drug for gout treatment, mainly exerting its effects by inhibiting microtubule polymerization and depolymerization, thus suppressing the inflammatory

response [14]. Colchicine has been demonstrated to regulate macrophage polarization, although its specific mechanism is not yet fully understood [13,15]. Therefore, uncovering the regulatory mechanism of colchicine on macrophage polarization is of great substantial for further understanding the pathophysiological processes of gout and developing new therapeutic strategies.

To explore the mechanisms underlying the improvement of gout inflammation by colchicine, this study employed R4F-NM@F127 nanoparticles as carriers to encapsulate colchicine and established mouse models of hyperuricemia and gout. Single-cell RNA sequencing (scRNA-seq) technology was utilized to reveal the heterogeneity of macrophages and the functional differences between M1 and M2 macrophages. Network pharmacology screening and molecular docking experiments confirmed the binding relationship between colchicine and key genes associated with macrophage polarization. Lentiviral intervention experiments were performed to validate the impact of these key genes on gout mice. Furthermore, we prepared neutrophil membrane (NM)-coated nanoparticles containing colchicine and investigated their therapeutic effect on gout mice.

The ultimate goal of this study is to elucidate the mechanism by which colchicine regulates gout inflammation through binding to key genes associated with macrophage polarization and to evaluate the therapeutic efficacy of colchicine loaded in R4F-NM@F127 nanoparticles. By unraveling the regulatory mechanisms of macrophage polarization, this study aims to provide new insights and approaches for the treatment of gout. Additionally, by utilizing R4F-NM@F127 nanoparticles as carriers for colchicine, this study also aims to offer a targeted therapeutic drug to alleviate gout symptoms and minimize toxicity to normal tissues.

Materials and Methods

Animal source and ethical statement

In this study, male C57BL/6 mice, 6 weeks old and weighing between 18 and 22 g, were obtained from Beijing Vital River Laboratory Animal Technology Co. Ltd. The mice were housed in a pathogen-free barrier facility with environmental control, including a 12-h light–dark cycle, temperature maintained at 24 ± 1 °C, and humidity between 50 ± 10%. Food and water were provided ad libitum [16].

All animal experiments in this study followed the relevant regulations and guidelines of our institutional Animal Ethics Committee and were approved by them. We made efforts to minimize pain and discomfort for all animals involved and to minimize the number of animals required for the experiments. Animals were handled and maintained in accordance with internationally recognized standards for animal welfare. Adequate care was provided for all animals, and appropriate measures were taken for their welfare and placement after the experiments concluded.

Animal therapy and grouping

Six groups of mice (5 mice per group) were randomly assigned: Control group, Model group, sh-NC group, sh-AHNAK group, Col group, and R4F-NM@F127-Col group. Prior to establishing a hyperuricemia and gout mouse model, gene knockdown was performed on the sh-NC and sh-AHNAK groups through tail vein injection of lentivirus. Specifically, the sequence for sh-NC was 5′-CCTAAGGTTAAGTCGCCCTCG-3′, the sequence for sh-AHNAK-1 was 5′-GCTTGAAGTTGCACCGTAAAG-3′, the sequence for sh-AHNAK-2 was 5′-GGTTCTGAACACGGTACAACC-3′, and the sequence for sh-AHNAK-3 was 5′-GGATGATGGAGTCTTTGTTCA-3′. The injection volume was 100 μl, and the lentivirus titer was 1×10^8 plaque-forming units (PFU)/ml. The design and synthesis of the lentiviral vectors were carried out by Guangzhou Ribo Biotechnology Co. Ltd. Subsequently, synovial fluid was extracted from the mouse joints, and the knockdown effect of sh-AHNAK was confirmed by reverse transcription quantitative polymerase chain reaction (RT-qPCR), selecting the most efficient knockdown sequence, sh-AHNAK-1, for further experiments (Fig. S1A) [17].

Establishment of hyperuricemia and gout mouse models

The model construction method followed previous research with some modifications [18]. From day 0 to day 24, excluding the Control group, mice were orally administered 250 mg/kg potassium oxonate (PO; 156124, Sigma-Aldrich, St. Louis, MO, USA) daily via gavage. On day 21 before surgery, mice were anesthetized by intraperitoneal injection of 3% isoflurane (PHR2874, Sigma-Aldrich, St. Louis, MO, USA). The experimental mice were then positioned supine, and the right hind limb was disinfected with iodine solution from the calf joint. Using a syringe inserted into the ankle joint cavity from the lateral aspect of the right hind foot at a 45° angle, the gout model group was injected with 50 μl of 10 mg/kg monosodium urate (MSU) crystal suspension (U2875, Sigma-Aldrich, St. Louis, MO, USA), while the Control group received 50 μl of phosphate-buffered saline (PBS; P2272, Sigma-Aldrich, St. Louis, MO, USA). Ankle joint diameter was measured with a caliper, and joint swelling was observed. Upon successful preparation, mice were intravenously injected with 5 mg/kg colchicine (Col group, HY-16569, MedChemExpress, USA) or R4F-NM@F127-Col (R4F-NM@F127-Col group) via the tail vein, while the other groups received PBS for subsequent experiments. On day 24, mice were euthanized under anesthesia for blood collection by cardiac puncture. Urine samples were also collected from each metabolic cage, centrifuged at 2,000*g* for 5 min for supernatant retrieval, and analyzed for uric acid and creatinine levels in serum and urine. Kidneys and ankle joints were excised and fixed with 4% paraformaldehyde (158127, Sigma-Aldrich, St. Louis, MO, USA) for further processing.

Measurement of urine uric acid and creatinine in mice

According to the manufacturer's instructions, serum and urine uric acid concentrations were determined using the Elabscience Uric Acid (UA) Colorimetric Assay Kit (E-BC-K016-M, Elabscience, Wuhan, China); serum and urine creatinine levels were measured using the Creatinine (Cr) Colorimetric Assay Kit (E-BC-K188-M, Elabscience, Wuhan, China); and blood urea nitrogen (BUN) levels were determined using the Urea Colorimetric Assay Kit (E-BC-K183-M, Elabscience, Wuhan, China). The formula for calculating the fractional excretion of uric acid (FEUA) is as follows: FEUA = (Serum creatinine × Urine uric acid)/(Urine creatinine × Serum uric acid) × 100 [18].

Xanthine oxidase activity in mouse liver

After obtaining liver tissue from the surgery, it was placed in a cold saline solution, homogenized, and then centrifuged at 10,000*g* at 4 °C for 10 min. Following the manufacturer's guidelines, xanthine oxidase (XOD) activity in the supernatant was measured using the XOD activity assay kit (BC1095, Solarbio, Beijing, China) [18].

H&E staining

The mice in each group were anesthetized with 3% isoflurane. The chest was opened, and 0.9% NaCl (IN9000, Solarbio, Beijing, China) was rapidly perfused through the left ventricle and aortic arch until all blood was completely drained. Next, a solution of 4% paraformaldehyde in 0.01 M PBS was perfused. After the limbs of the mice became stiff, the right kidney and ankle joint were removed and fixed in the same 4% paraformaldehyde solution for 24 h. Subsequently, dehydration, clearing, and routine paraffin embedding were performed using a graded ethanol series. The kidney tissue was sliced into 3-μm sections using a microtome, while the joint sections were 5 μm thick after decalcification. The slices were baked at 60 °C for 1 h and then dewaxed with xylene (247642, Sigma-Aldrich, St. Louis, MO, USA). After hydration, routine hematoxylin and eosin (H&E) staining was performed. The sections were stained with hematoxylin (HHS32, Sigma-Aldrich, St. Louis, MO, USA) for 2 min, rinsed with tap water for 10 s, and differentiated in 1% hydrochloric acid ethanol for 10 s. After brief washing with distilled water for 1 min, the sections were stained with eosin (230251, Sigma-Aldrich, St. Louis, MO, USA) for 1 min, rinsed with distilled water for 10 s, and then dehydrated with a graded ethanol series and cleared with xylene. Finally, the sections were mounted in neutral resin. The tissue morphology was observed using an XP-330 optical microscope provided by Shanghai Bingyu Optical Instrument Co. Ltd. [19].

PBMC sample preparation

Collection of peripheral blood from control and experimental mice ($n = 3$) was done with 2 ml of blood added into EDTA anticoagulant tubes and diluted with 2 ml of 1× PBS. Ficoll lymphocyte separation solution (P8900, Solarbio) of equal volume was added to a 50-ml centrifuge tube and centrifuged at 700*g* at 20 °C for 20 min. The cell layer was carefully transferred to a 15-ml centrifuge tube. The cell layer was washed with 10 ml of 1× PBS and centrifuged at room temperature at 300*g* for 10 min. The supernatant was discarded, and the cells were suspended in 1 ml of RPMI 1640 medium (R8758, Sigma-Aldrich, St. Louis, MO, USA) containing 0.04% bovine serum albumin (BSA; B2064, Sigma-Aldrich, St. Louis, MO, USA).

The Zombie NIR Fixable Viability Kit (423105, BioLegend, San Diego, USA) was used for live/dead discrimination. Fluorescence-activated cell sorting (FACS) was performed using a BD FACS Aria II cell sorter (BD, USA) to sort the cells. The cells were sorted into capture medium (PBS containing 0.04% BSA). Single-cell samples were counted using the Countess II automated cell counter and trypan blue cell counting. When cell viability exceeded 80%, the cells were loaded onto the 10x Genomics Chromium chip following the manufacturer's instructions [20].

Single-cell RNA sequencing

The gel bead emulsion (GEM) was formed by mixing single-cell suspension, gel beads, and oil using the 10x Genomics Chromium controller. After droplet formation, the samples were added to PCR tubes, subjected to reverse transcription on a T100 Thermal Cycler (Bio-Rad), and incubated at 53 °C for 45 min, 85 °C for 5 min, followed by cooling at 4 °C. The complementary DNA (cDNA) was generated and amplified, and its quality was assessed using the Agilent Bioanalyzer 2100. Libraries were constructed by adding P5 and P7 primers, Read 2 (reads 2 sequencing primer site), and Sample Index and then underwent quality control (QC) before being sequenced on the Illumina HiSeq4000 PE125 platform.

Subsequently, the acquired libraries were further analyzed using 10x Cell Ranger (version 2.2.0) for scRNA-seq data. The base-call files (BCLs) from the Illumina sequencer were converted to FASTQ files, followed by alignment, filtering, barcode assignment, and counting unique molecular identifiers (UMIs). The reads were aligned to the mouse reference transcriptome (mm10) using STAR (Spliced Transcripts Alignment to a Reference). Primary QC was performed using Cell Ranger to generate high-quality data [20].

Single-cell transcriptomic analysis

The single-cell data were analyzed using the "Seurat" package in R software. QC was performed based on the criteria of nFeature_RNA > 200 and nFeature_RNA < 5,000. To reduce the dimensionality of the scRNA-seq dataset, principal components analysis (PCA) based on variance was employed, selecting the top 4,000 highly variable genes. The Elbowplot and JackStrawPlot functions provided by the Seurat package were used to determine the appropriate number of principal components for downstream analysis. The FindClusters function in Seurat was then applied for cell clustering identification with a default resolution value (res = 1). Subsequently, the t-stochastic neighborhood embedding (tSNE) algorithm was used for dimensionality reduction of the scRNA-seq data. The Seurat package was used to identify marker genes for each cell subset. Finally, the "SingleR" package, in conjunction with the online database CellMarker, was employed for cell annotation and extraction of the target cell population for reclustering analysis. The "CellChat" package was used for cell communication analysis, while the "Monocle2" package was utilized for pseudo-time analysis. The "EnhancedVolcano" package was used to visualize differential genes in M2 macrophages between the Control and Model groups [21].

Acquisition of colchicine's target sites

To obtain the target sites of colchicine, we utilized the Batman database (http://bionet.ncpsb.org.cn/batman-tcm/#/home), the comparative toxicogenomics database (CTD) (https://ctdbase.org/), and the SwissTargetPrediction database (http://www.swisstargetprediction.ch/index.php). To eliminate duplicates [22], we employed these resources to identify the target sites.

Identification of gout-related targets

The relevant targets were searched in the GeneCards database (https://www.genecards.org/), CTD database, and Harmonizome 3.0 database (https://maayanlab.cloud/Harmonizome/) using "Gout" as a keyword. The retrieved targets were integrated, duplicates were removed, and a disease-related target database was created [23].

Molecular docking

The protein structure of the target protein AHNAK was downloaded from the Protein Data Bank (PDB) database (https://www.rcsb.org/). The 2-dimensional (2D) structure of the colchicine compound was downloaded from the PubChem database (https://pubchem.ncbi.nlm.nih.gov/). The compound structure was then converted into a 3D structure using the Chem3DUltra 14.0 software and subjected to energy minimization using the MM2 algorithm. Next, the AHNAK protein was processed by dehydration and removal of organic compounds using the PyMOL software. The AHNAK protein was processed further by hydrogenation and charge calculation using AutoDockTools 1.5.6. The compound and the target protein receptor were converted into "pdbqt" files, and suitable protein binding sites were identified. Finally, molecular docking was performed using Vina 1.1.2 to evaluate the docking energy values [24].

Surface plasmon resonance experiment

The equilibrium binding constant (K_D) between colchicine and AHNAK was determined using an Open surface plasmon resonance (SPR) assay on a BIAcore 4000 instrument (BIAcore). In brief, the AHNAK protein (10 mg/ml, ab163959, Abcam) was covalently immobilized on a COOH sensor chip using the 1-ethyl-3-(3-dimethylaminopropyl)carbodiimide/*N*-hydroxysuccinimide (EDC/NHS) chemical method with 200 μl of AHNAK protein. Subsequently, colchicine was serially diluted to various concentrations using a running buffer and injected onto the chip starting from low to high concentrations. During each cycle, a constant flow rate of 20 μl/min was used to pass 200 μl of the sample through the chip for 4 to 6 min. Following detection, 0.05% sodium dodecyl sulfate (SDS) was added to separate the peptide from the target protein. Lastly, the kinetic parameters of the binding reaction were determined using Trace Drawer software (Ridgeview Instruments AB, Kingdom of Sweden) [25].

Isolation and identification of peripheral neutrophils

Peripheral neutrophils were activated in mice by intraperitoneal injection of 1.5 mg/kg lipopolysaccharide (LPS; L8880, Solarbio, Beijing, China). After 6 h, peripheral blood was collected from the mice into tubes containing EDTA-2K as an anticoagulant. The mice's peripheral blood neutrophils were isolated using the Mouse Peripheral Blood Neutrophil Isolation Kit (P9201, Solarbio, Beijing, China). Subsequently, the isolated neutrophils were resuspended in PBS and stored at −80 °C for subsequent membrane isolation. The cell suspension was further confirmed for neutrophil identification using Giemsa staining, and their morphology was examined using Olympus BX53 (Japan). Neutrophils exhibited characteristic features of granules and multi-lobed nuclei (Fig. S1B and C) [26].

Isolation of NMs

The frozen neutrophil cell suspension was thawed and washed 3 times with PBS (800*g* centrifugation). Subsequently, the neutrophil cells were suspended in a hypotonic lysis buffer containing 225 mM D-mannitol (IM0040, Solarbio, Beijing, China), 30 mM tris–HCl (pH 7.5, T1140, Solarbio, Beijing, China), 75 mM sucrose (S8271, Solarbio, Beijing, China), 0.2 mM EGTA (E8050, Solarbio, Beijing, China), and a cocktail of protease inhibitors (Cocktail, HY-K0010, MedChemExpress, USA). The neutrophil cells were disrupted using a Dounce homogenizer (64790-01, Biosharp, Beijing, China) with a grinding pestle (40 times). The homogenized solution was then centrifuged at 20,000*g* for 25 min at 4 °C. The supernatant was further centrifuged at 120,000*g* for 60 min at 4 °C. The resulting NMs, as the granules, were collected and stored at −80 °C for later use [26].

Synthesis and characterization of R4F-NM@F127 and R4F-NM@F127-Col

Pluronic F127 (ST501, Beyotime, Shanghai, China) or Pluronic F127-Colchicine (Pluronic F127-Col) polymer prepared using the thin-film hydration method was mixed with NMs. Subsequently, a liposome extruder was used to pass the mixture through polycarbonate membranes with pore sizes of 400 and 100 nm, respectively, for 20 cycles to obtain NM@F127 nanoparticles or NM@F127-Col nanoparticles. Subsequently, NM@F127 and NM@F127-Col were functionalized with R4F peptide by dissolving R4F peptide in deionized water, dropwise addition to the aforementioned mixture, and overnight storage at 4 °C. Unincorporated peptides were removed by centrifugation (200*g*, 20 min), enabling the binding of R4F peptide to the phospholipids on NMs (https://doi.org/10.1016/j.nantod.2023.101864), resulting in the preparation of R4F-NM@F127 and R4F-NM@F127-Col. The average particle size distribution, zeta potential, and PDI of R4F-NM@F127 and R4F-NM@F127-Col were determined using a Malvern particle size analyzer (Zetasizer Nano ZS90, Malvern, UK). The morphology of R4F-NM@F127 and R4F-NM@F127-Col was examined by transmission electron microscopy (TEM). The absorption wavelength of DiR-BOA (1,1′-dioctadecyl-3,3,3′,3′-tetramethylindotricarbocyanine iodide bisoleate) loaded onto R4F-NM@F127 and R4F-NM@F127-Col was measured using an ultraviolet-visible (UV-Vis) spectrophotometer [26].

Toxicological evaluation

Serum toxicity biomarkers, aspartate transaminase, and alanine transaminase were measured using the aspartate aminotransferase (AST) activity assay kit (MAK055, Sigma-Aldrich, St. Louis, MO, USA) and the alanine aminotransferase (ALT) activity assay kit (MAK052, Sigma-Aldrich, St. Louis, MO, USA), respectively. Tissue histopathology was performed on the heart, liver, spleen, lungs, kidneys, and brain. Tissue samples were fixed in 4% paraformaldehyde, embedded in paraffin, and stained with H&E, and all images were captured and analyzed under an Olympus BX53 microscope (Japan) [26].

In vitro drug release study

Colchicine standard was placed in a 100-ml volumetric flask, dissolved in analytical grade methanol, and diluted to the mark. A specific volume of the standard solution was transferred to an Eppendorf tube and made up to 1 ml with methanol solution. After mixing by pipetting, the sample was analyzed using high-performance liquid chromatography (HPLC). The release of cells from inflammation in R4F-NM@F127-Col was determined using a dialysis method.

The prepared R4F-NM@F127-Col solution was transferred to a 14-kDa molecular weight cutoff dialysis bag (MP1719-5M, Shanghai MaoKang Biotechnology Co. Ltd., China) and placed in 1 l of 0.1% SDS phosphate buffer (pH 7.4) with stirring at room temperature. At multiple time points, 50 μl of the R4F-NM@

F127-Col solution was extracted from the dialysis bag, while 50 µl of PBS was added to the dialysis bag. The extracted R4F-NM@F127-Col solution was diluted with methanol solution to fall within the linear range of the established standard curve, and the drug release rate was determined by HPLC [27].

High-performance liquid chromatography

HPLC was utilized to investigate the linear correlation between colchicine concentration and peak area. The chromatographic conditions for colchicine measurement were as follows: mobile phase of methanol/water = 65/35 (v/v), sample volume of 5 µl, flow rate of 1.0 ml/min, column temperature of 50 °C, and detection wavelength of 350 nm. The encapsulation efficiency of R4F-NM@F127-Col was calculated as follows: The encapsulation rate was obtained by dividing the total drug added amount by the drug amount not included in the encapsulation, and the drug loading was determined by dividing the total drug carried by the total weight of the nanoparticles.

Degradation curve generation:

A microtube was filled with 1 ml of standard culture medium, and weight loss of recovered R4F-NM@F127-Col samples was measured at various time intervals (ranging from 1 h to 1 week). At each time point, the samples were centrifuged at 10,000*g* for 5 min at room temperature, dried, and then weighed. The microtubes were subsequently stored at 37 °C. The degradation curve was evaluated by measuring the weight loss percentage at each time interval.

In vivo fluorescence imaging

Nine gout mice were randomly divided into 3 groups (3 mice per group). The mice were intravenously injected with 100 µl of sterile DiR-BOA-labeled R4F-NM@F127, F127, and an equal amount of DiR-BOA for the Control group via the tail vein. Imaging was conducted at 1, 3, 6, 12, and 24 h using the small animal in vivo imaging system (CLS136340, IVIS Lumina XRMS, PerkinElmer, Waltham, USA). The mice were euthanized at 24 h, and fluorescence imaging was performed on the heart, liver, spleen, lungs, kidneys, brain, and joints. The average fluorescence intensity was measured using live imaging software to quantitatively analyze the tissue distribution of R4F-NM@F127 [27].

Cell culture and treatment

Mouse RAW264.7 cells (TIB-71) were obtained from the American Type Culture Collection (ATCC) and cultured in Dulbecco's modified Eagle's medium (DMEM) (30-2002, ATCC, USA) supplemented with 10% fetal bovine serum (164210, Procell, Wuhan, China) and 1% penicillin-streptomycin solution (PB180120, Procell, Wuhan, China). The cells were cultured at 37 °C in a humidified atmosphere containing 5% CO_2 and 95% air.

RAW264.7 cells were seeded in 6-well plates at a density of 1×10^6 cells/ml and divided into the following groups: Normal, LPS, Col, and R4F-NM@F127-Col. After overnight incubation, the cells in the LPS group were treated with 100 ng/ml LPS for 30 min. Subsequently, the culture medium was supplemented with either 0.25 or 0.5 µM colchicine or R4F-NM@F127-Col, and the cells were further incubated for 6 h. The Normal group and LPS group were given an equal volume of culture medium [26].

Cell viability assay

RAW264.7 cells were seeded at a density of 5×10^3 cells per well in a 96-well plate, with 4 replicate wells per group. When the cell confluence reached approximately 50%, different concentrations of R4F-NM@F127-Col or colchicine were added, and the cells were incubated for 24 h. After that, the cells were treated with MTS [3-(4,5-dimethylthiazol-2-yl)-5-(3-carboxymethoxyphenyl)-2-(4-sulfophenyl)-2*H*-tetrazolium] reagent (ab197010, Abcam, USA) for 4 h, and the absorbance at 490 nm was measured. Cells with higher viability emitted stronger fluorescence, while cells with lower viability emitted weaker fluorescence. Dead cells did not emit any fluorescence. The calculation formula for cell viability was as follows: (total cell count − dead cell count)/total cell count × 100% [27].

Flow cytometry

To assess M1/M2 polarization, RAW264.7 cells were seeded in a 96-well plate with a density of 1.5×10^4 cells per well and incubated overnight in a culture medium. Except for the Normal group, the other groups were treated with LPS (100 ng/ml) for 30 min. Then, the cells were treated with either colchicine or R4F-NM@F127-Col in the culture medium for 24 h. After incubation, the RAW264.7 cells were blocked with CD16/32 (553141, BD Biosciences, USA) for 10 min to prevent Fc receptor binding and then stained with allophycocyanin (APC) anti-mouse CD86 antibody (ab134385, Abcam, USA) and peridinin chlorophyll protein (PerCP)/Cyanine5.5 anti-mouse CD206 antibody (141716, BioLegend, San Diego, USA) for 30 min. Flow cytometry analysis was performed. For macrophages in joint fluid, sterile PBS was injected into the joint cavity using a syringe, and fluid was extracted from the mice. The cells were filtered through a 70-µm cell strainer and washed once with PBS to obtain a single-cell suspension. The single-cell suspension was blocked with CD16/32 for 10 min to prevent Fc receptor binding and then stained with APC anti-mouse CD86 antibody and PerCP/Cyanine5.5 anti-mouse CD206 antibody for 30 min. Flow cytometry was used to detect the cells. FlowJo software was used for data analysis [26].

Immunofluorescence staining

Immunofluorescence staining of the synovial membrane of the knee joint was performed as follows: Freshly collected knee joints were fixed in 4% paraformaldehyde for 24 h. Subsequently, they were decalcified in PBS containing 15% EDTA at 4 °C for 3 days with daily replacement of the decalcification solution. After decalcification, the samples were washed overnight at 4 °C. The tissues were then protected by 30% sucrose in PBS at 4 °C until they sank and were embedded in optimal cutting temperature compound (OCT) compound, followed by freezing on dry ice. Tissue sections of 10-µm thickness were cut using a cryostat (Dako Biotechnology Co. Ltd., China) and mounted on poly-L-lysine-coated glass slides. After blocking with 1% BSA in the blocking buffer for 1 h, the sections were incubated overnight at 4 °C with anti-F4/80 antibody (ab6640, 5 µg/ml, Abcam). Following incubation, the tissue sections were washed 3 times with PBS and incubated in the dark at room temperature for 1 h with Goat Anti-Rabbit IgG H&L (Alexa Fluor 594) secondary antibody (ab150080, 1:1,000, Abcam). Nuclei were counterstained with 4′,6-diamidino-2-phenylindole (DAPI). The slices were observed using an Olympus Fluorview-3000 confocal microscope (Olympus Optical Ltd., Japan), and the data were analyzed using ImageJ software [26].

RT-qPCR

Total RNA was extracted from both tissue and cells using the Thermo Fisher Tissue RNA Extraction Kit (12183018A, USA) and Cell RNA Extraction Kit (12183020, USA) following the manufacturer's instructions. For cDNA synthesis, 1 μg of total RNA was reverse transcribed into cDNA using the First Strand cDNA Synthesis Kit (K1622) from Thermo Fisher. The synthesized cDNA was then subjected to RT-qPCR analysis using the Fast SYBR Green PCR Kit (4385610, Thermo Fisher, USA) from Applied Biosystems and the ABI PRISM 7500 RT-PCR system (4351104, Thermo Fisher, USA). Each well was run in triplicate. The relative expression levels of mRNA were analyzed using the $2^{-\Delta\Delta CT}$ method, where $\Delta\Delta Ct$ = (mean Ct value of target gene in experimental group − mean Ct value of reference gene in experimental group) − (mean Ct value of target gene in control group − mean Ct value of reference gene in control group). Glyceraldehyde-3-phosphate dehydrogenase (GAPDH) was used as the reference gene. The RT-qPCR was performed on the StepOnePlus system, with a cycling protocol of 1 cycle at 95 °C for 15 min, followed by 40 cycles of 95 °C for 10 s and 60 °C for 60 s [28]. The primer sequences used in the experiment are listed in Table S1. All reagents and materials used in the experiment were purchased from Wuhan Saivell Biological Technology Co. Ltd.

Western blot

Proteins were extracted from mouse serum and macrophages using the Serum Protein Extraction Kit (EX1180) and the Cell Protein Extraction Kit (EX2170) from Solarbio, Wuhan. The protein concentration was determined using the BCA Protein Assay Kit (BCA1-1KT) from Sigma-Aldrich. Equal amounts of protein (20 μg per lane) were subjected to 10 to 12% SDS–polyacrylamide gel electrophoresis (PAGE) electrophoresis and transferred onto a polyvinylidene difluoride membrane from EMD Millipore. Subsequently, the membrane was blocked with 5% BSA for 2 h, washed with PBS, and incubated overnight at 4 °C with the primary antibodies (see Table S2 for detailed information on the primary antibodies). After washing, the membrane was then incubated at room temperature for 2 h with the secondary antibody (goat anti-rabbit horseradish peroxidase conjugate, ab6721, 1:2,000) from Abcam, followed by visualization using the iBright FL1500 Enhanced Chemiluminescence System from Thermo Fisher. The anti-GAPDH antibody (ab9485, 1:2,500) from Abcam was used as an internal control [29]. The experiment was performed in triplicate.

Statistical analysis

All data were processed using IBM SPSS Statistics (Chicago, IL, USA) version 21.0. Descriptive statistics were reported as mean ± standard deviation. Independent sample *t* test was used to compare differences between 2 groups, while one-way analysis of variance (ANOVA) followed by Tukey's post hoc test was employed for comparisons among multiple groups. Differences were considered statistically significant at $P < 0.05$.

Results

Successful modeling of hyperuricemia and gout mouse model

We established hyperuricemia and gout mouse models using chronic PO exposure and MSU subcutaneous injection [18]. PO was orally administered once daily from day 0 to day 21. On day 21, each mouse received an injection of 50 μl of MSU (50 μl, 10 mg/kg) into the right tibiotarsal joint (ankle joint) (Fig. 1A). Quantitative analysis of joint swelling using a caliper revealed significant swelling in the right joints of the model mice compared to the control group (Fig. 1B and C). Subsequently, levels of uric acid, creatinine, and BUN in mouse serum and urine were measured. The results showed a significant increase in serum uric acid, creatinine, and BUN levels, while urinary uric acid and creatinine levels were significantly decreased, and FEUA values were significantly reduced in the Model group compared to the control group (Table S3). XOD activity in the liver, as determined by liver XOD assay, was approximately 23 nmol/min in the control group. The model group exhibited a significant 2.5-fold increase in liver XOD activity compared to the control group (Fig. 1D). Serum cytokine expression was detected by RT-qPCR, and the results showed a significant increase in tumor necrosis factor-α (TNF-α), IL-1β, IL-6, and IL-12 levels and a significant decrease in IL-10 levels in the serum of the model group mice compared to the control group (Fig. 1E). H&E staining revealed inflammatory cell infiltration in the renal tubular interstitium, severe tubular dilation, and vacuolization of renal tubular epithelial cells in the model group mice (Fig. 1F). The synovial cells of the knee joint displayed varying degrees of necrosis, synovial tissue congestion, and inflammatory cell infiltration (Fig. 1G).

Modulation of immune activity through ligand–receptor interactions by myeloid cells during the process of gout development

To investigate the molecular mechanisms underlying gout development, we performed single-cell sequencing of peripheral blood mononuclear cells (PBMCs) from Model and Control mice (Fig. 2A). We obtained scRNA-seq datasets and integrated the data using the Seurat package. Initially, we examined the scRNA-seq data from the Model and Control groups. Prior to filtering, we assessed the number of genes (nFeature_RNA), mRNA molecules (nCount_RNA), and percentage of mitochondrial genes (percent.mt) in all cells. The results showed that cells with nFeature_RNA < 8,000, nCount_RNA < 50,000, and percent.mt = 0 (Fig. S2A) were considered low-quality cells and were excluded. This filtering step resulted in an expression matrix consisting of 19,833 genes and 17,839 cells. After filtering, the nFeature_RNA, nCount_RNA, and percent.mt of cells in each group are presented in Fig. S2B. The correlation coefficients between nCount_RNA and percent.mt ($r = -0.02$) and nCount_RNA and nFeature_RNA ($r = 0.94$) of the filtered data are shown in Fig. S2C, indicating good data quality postfiltering for subsequent analysis. The integration of data from the Model and Control groups was performed using the "FindIntegrationAnchors" function, followed by further analysis of the filtered cells. Highly variable genes were selected based on gene expression variance, and the top 4,000 genes with significant variance were chosen for downstream analysis (Fig. S2D).

Subsequently, PCA was employed to linearly reduce the dimensionality of the data, revealing the presence of batch effects among the samples (Fig. S3A). To eliminate batch effects, the harmony package was utilized for batch correction of the sample data. The results after correction showed successful elimination of batch effects between the samples (Fig. S3B). We selectively displayed the major constituent genes in the top 4

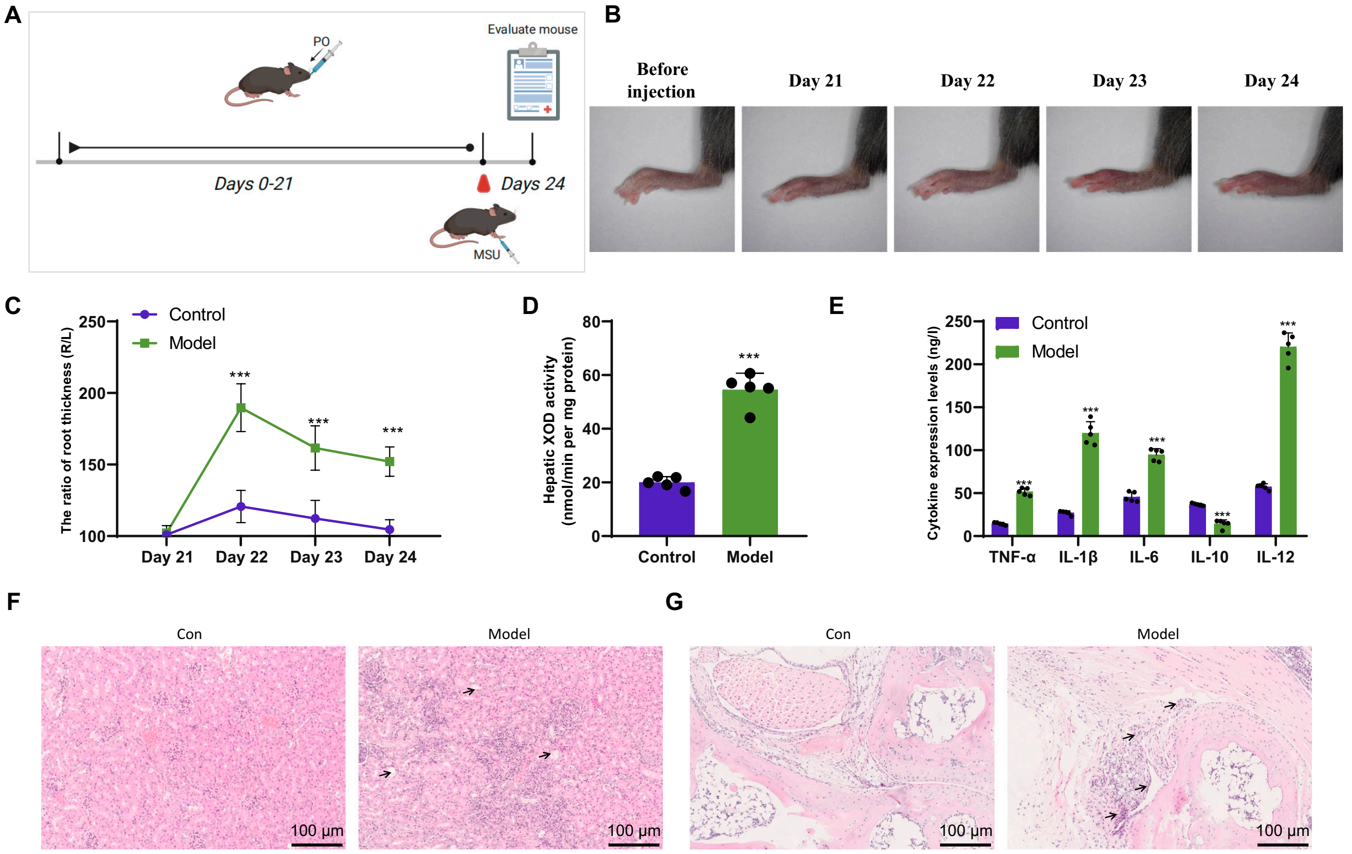

Fig. 1. Construction and validation of mouse models for hyperuricemia and gout induced by PO and MSU. (A) Schematic diagram of the preparation of mouse models for hyperuricemia and gout induced by PO and MSU. (B) Representative ankle joint photos of mice before and 4 days after model establishment. (C) Measurement of ankle joint swelling ratio in each group of mice. (D) Measurement of XOD activity in the liver of mice in each group. (E) Enzyme-linked immunosorbent assay (ELISA) experiment to detect cytokine expression levels in each group of mice. (F and G) H&E staining analysis of kidney (F) and ankle joint (G) tissue pathology in each group of mice. Black arrows indicate cell vacuolation, synovial hyperplasia, etc. (scale bar, 100 μm). Comparison with the Control group, $P < 0.05$. Comparison with the Control group, $P < 0.01$. **Comparison with the Control group, $P < 0.001$. $N = 5$.

principal components (PCs) (Fig. S3C), and a heatmap of the top 4 PCs was generated using the "DimHeatmap" function (Fig. S3D). Additionally, an ElbowPlot was used to rank the standard deviation of the PCs, indicating that PC1 to PC20 adequately represented the information contained in the selected highly variable genes and possessed meaningful analytical substantial (Fig. S3E).

We visualized the first 20 PCs using the "JackStrawPlot" function, comparing the distribution of *P* values and mean values for each PC. Typically, PCs with smaller *P* values are considered "important" (indicated by solid lines above the dashed line), as they reflect the information contained in highly variable genes identified in previous filtering steps. We performed tSNE analysis on the top 6 PCs with a *P* value of <0.05 (Fig. S3F). After tSNE clustering analysis, we assigned all cells to 15 cell clusters (Fig. S4A) and obtained the characteristic genes for each cluster. We further generated expression profiles for the top 10 differentially expressed genes specific to each of these 15 cell clusters (Table S4 and Fig. S4B). Through clustering analysis, we observed cells from each sample distributed across multiple clusters, with noticeable distinctions between the Model and Control groups (Fig. 2B). Using the Bioconductor/R package "SingleR", we annotated the marker genes of these 15 cell clusters and categorized them into 6 cell types: T cells, neutrophils, natural killer cells, myeloid cells, B cells, and erythroid cells (Fig. 2C). Additionally, we visualized the cell composition proportions between the Model and Control groups. The results indicated a higher proportion of basophils, myeloid cells, and neutrophils in the Model group compared to the Control group (Fig. 2D and Fig. S4C).

Next, we utilized the R package "CellChat" to investigate the pathway activities between different cell types in the Control and Model groups. First, we displayed a comparison of communication numbers and intensities between the Control and Model groups (Fig. 2E) and identified and visualized conserved and specific signaling pathways between these 2 groups (Fig. 2F). We found that macrophage migration inhibitory factor (MIF) was present in both the Control and Model groups but showed increased activity in the Model group. MIF acts as an upstream regulator for both adaptive and innate immunity; however, when dysregulated, it becomes a critical driving factor for acute or chronic inflammation and plays a role in leukocyte recruitment [30]. Differential analysis revealed that LGALS9_CD45 and LGALS9_CD44 were key ligand–receptor interactions between myeloid cells and other immune cells (Fig. S5).

The significant role and regulatory mechanisms of macrophages in the onset of gout

We performed a reclustering analysis on the myeloid cell population. Here, we selectively display the major gene composition

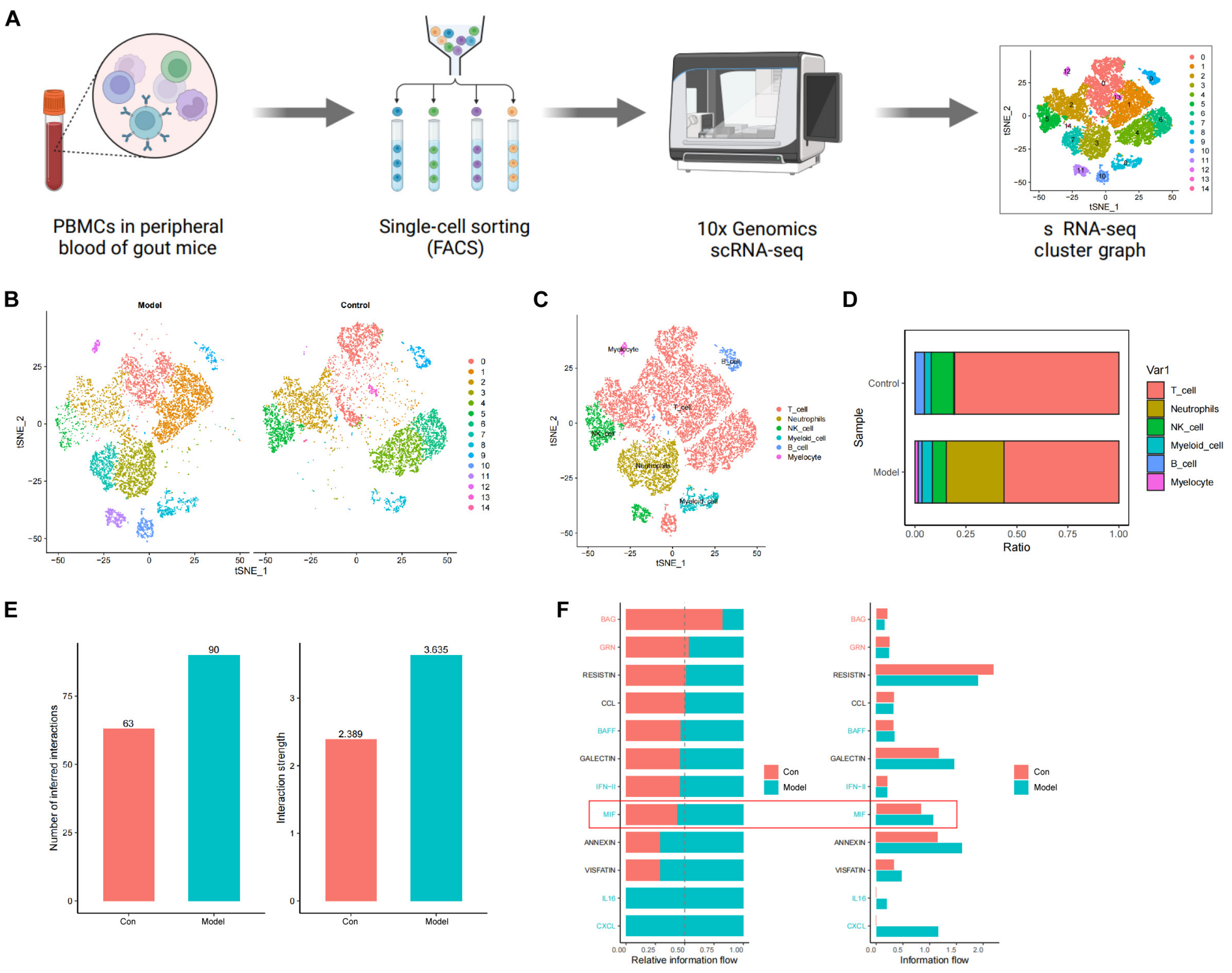

Fig. 2. Revealing cellular heterogeneity in the pathogenesis of gout. (A) Workflow diagram of single-cell sequencing. (B) tSNE clustering analysis of samples from the Control group and Model group. (C) Annotation of 15 cell clusters into 6 cell types using the "SingleR" package. (D) Proportions of different cell subpopulations in the Control group and Model group, with cell subpopulations represented by different colors. (E) Comparison of communication quantity and intensity between the Control group and Model group. (F) Visualization of conserved and specific signaling pathways between the Control group and Model group.

(Fig. S6A) and the heatmap (Fig. S6B) of the top 4 PCs. Using the "JackStrawPlot" and "ElbowPlot" functions (Fig. S6C and D), we chose the first 15 PCs for tSNE analysis, resulting in the clustering of astrocytes into 6 distinct cell clusters (Fig. S6E). We identified characteristic genes for each cell cluster (Table S5) and generated expression profiles for the top 10 genes in the cluster-specific marker genes (Fig. S6F). Based on these cell cluster marker genes, we ultimately determined 3 types of cell clusters: cluster 0 as monocytes, cluster 1 as M2 macrophages, and cluster 3 as M1 macrophages (Fig. 3A), with the associated correlation dot plots between these cell types and the marker genes (Fig. 3B). Furthermore, we observed significant differences between the Control and Model groups in the myeloid cell population (Fig. S6G).

Subsequently, we conducted pseudotime analysis on individual cells using the "Monocle2" package. The cell transition trajectory displayed different clusters in the pseudotime, and we color-coded the pseudotime plot based on the originating tissues to identify the cellular composition. The cells exhibited a clear trajectory, indicating continuous changes in their states during differentiation or development (Fig. 3C), which were further divided into 6 states represented by different colors (Fig. 3D). Analyzing these states based on cell types, we found that early-stage aggregated cells were monocytes, which persisted throughout the differentiation or development process. M1 macrophages were predominantly located at the end of the time axis, while M2 macrophages were distributed in both intermediate and end stages (Fig. 3E).

To explore key genes regulating macrophage polarization, we conducted a differential gene expression analysis on M2 macrophages from the Control and Model groups, revealing 9 significantly up-regulated genes (UQCR11, MT-ND4L, CTSS, RPL13A, RPL28, AHNAK, MT-CO1, PSAP, and RPL7) in the Model group (Fig. 3F).

Mechanism of action of colchicine on gout inflammation and its interaction with AHNAK protein

Colchicine is an alkaloid extracted from the colchicum autumnale plant (Fig. 4A). To further investigate the target of colchicine

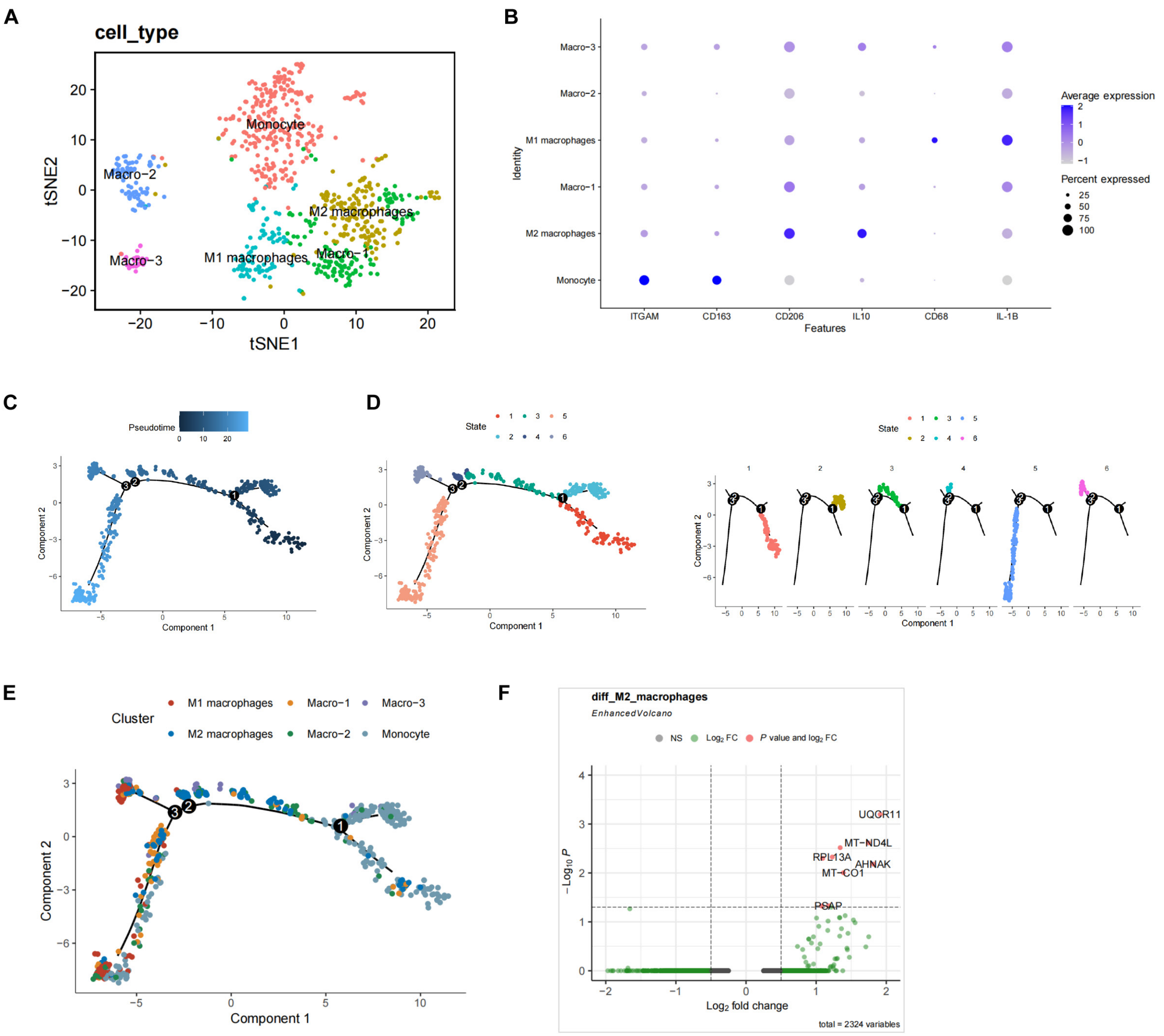

Fig. 3. Reclustering analysis of myeloid cells in scRNA-seq data. (A) Annotation of single-nucleus cells, M1 macrophages, and M2 macrophages in the 6 cell clusters based on cell marker genes. (B) tSNE plot of 3 cell marker genes. (C) Trajectory skeletal plot colored by pseudotime, with each point representing a cell. (D) Trajectory skeletal plot colored by state, with different states represented by different colors. (E) Trajectory skeletal plot colored by cell type. (F) Volcano plot of differentially expressed genes in M2 macrophages between the Control group and Model group, with red dots representing $|\log_2FC| > 0.5$ and $P < 0.05$, green dots representing $\log_2FC > 0.5$, and gray dots representing genes with no significant difference.

in gout inflammation, we retrieved its target proteins from the Batman database, CTD database, and SwissTargetPrediction database for screening. Ninety-nine target proteins were identified from the Batman database, 1,082 from the CTD database, and 101 from the SwissTargetPrediction database. After removing duplicates, a total of 1,231 target proteins of colchicine were obtained (Fig. 4B and Table S6).

Next, we retrieved gout-related genes from the Harmonizome 3.0 database, CTD database, and GeneCards database and further selected them. We found 71 targets from the Harmonizome 3.0 database, 17,514 targets from the CTD database, and 1,875 targets from the GeneCards database. After removing duplicates, we obtained a total of 18,042 genes related to gout (Fig. 4C and Table S7). By intersecting the target proteins of colchicine, gout-related genes, and core regulatory genes of macrophage polarization from the scRNA-seq dataset, we identified a crucial gene, AHNAK (Fig. 4D).

Furthermore, we performed molecular docking analysis of AHNAK and colchicine using software such as AutoDockTools 1.5.6 and Vina 1.1.2. The docking was repeated 3 times for both the molecule and protein, and the average of the lowest binding free energy was calculated. The molecular docking results of AHNAK and colchicine were visualized in 3D images (Fig. 4E), demonstrating the binding mode of the target protein receptor with the compound and its interaction with surrounding amino acid residues. Additionally, SPR indicated a high-affinity binding between colchicine and recombinant AHNAK protein (Fig. 4F).

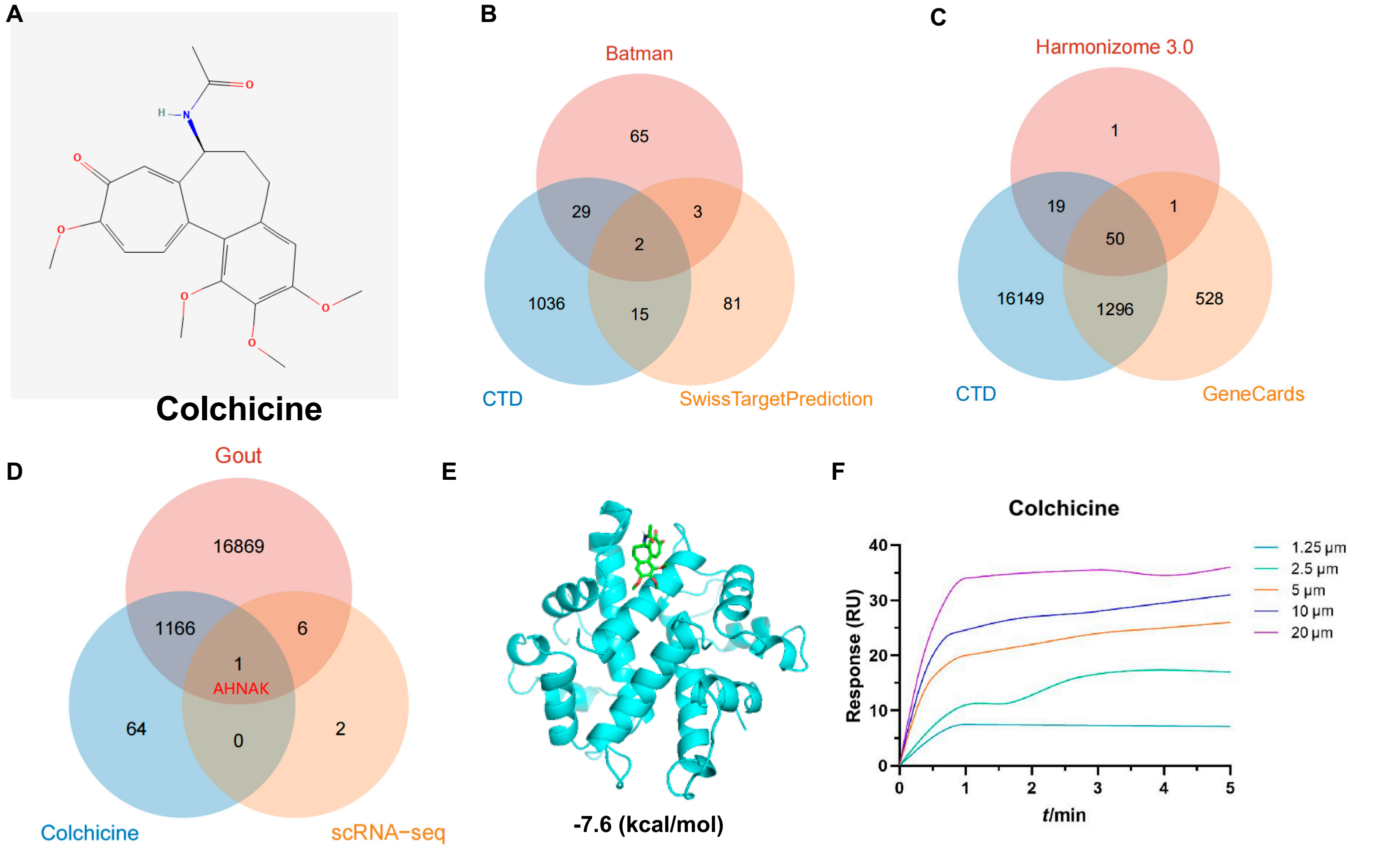

Fig. 4. Network pharmacology analysis for screening key genes. (A) Chemical structure of colchicine. (B) Venn diagram showing the number of targets of colchicine in 3 drug target databases. (C) Venn diagram showing the number of gout-related genes in 3 disease gene databases. (D) Venn diagram showing the intersection of gout-related genes and target proteins corresponding to colchicine. (E) Molecular docking of AHNAK with colchicine. Blue represents AHNAK protein secondary structure, and green represents colchicine. (F) SPR experiment evaluating the binding of AHNAK with colchicine (1.25, 2.5, 5, 10, and 20 μM, from bottom to top).

Alleviation of joint inflammation and uric acid generation in a gout mouse model through AHNAK underexpression

In order to verify the crucial role of AHNAK in gout, we intervened in AHNAK expression in mice using lentivirus carrying sh-AHNAK. Subsequently, we established experimental groups and control groups of mice by inducing hyperuricemia and gout using PO and MSU, respectively, naming them the sh-NC group and the sh-AHNAK group. First, we quantitatively analyzed the degree of joint swelling using a caliper. The results showed that there was no significant difference in the degree of right joint swelling in mice from the sh-NC group compared to the Model group, while the mice from the sh-AHNAK group displayed significant improvement in the degree of right joint swelling (Fig. 5A). Subsequently, we measured the levels of uric acid, creatinine, and BUN in mouse serum and urine. The results revealed that there were no differences in the levels of uric acid, creatinine, and BUN in the serum of mice from the sh-NC group compared to the Model group, as well as in the levels of uric acid and creatinine in the urine. However, the sh-AHNAK group showed significantly decreased levels of uric acid, creatinine, and BUN in the serum, along with significantly increased levels of uric acid, creatinine, and FEUA in the urine (Table S3). Additionally, the hepatic XOD activity results showed that the liver XOD activity of mice from the sh-NC group was similar to that of the Model group, while the sh-AHNAK group displayed significantly decreased liver XOD activity (Fig. 5B). Furthermore, histopathological staining using H&E revealed that mice from the sh-AHNAK group exhibited improved interstitial and knee joint inflammation, as well as alleviated synovial cell necrosis (Fig. 5C and D).

In further experiments, we extracted synovial tissue from mice in each group to investigate macrophage polarization. We used RT-qPCR to measure the expression levels of key biomarkers TNF-α, IL-1β, and inducible nitric oxide synthase (iNOS) mRNA for M1 macrophages and Arg-1 and IL-10 mRNA for M2 macrophages. The results showed that compared to the Control group, the expression levels of TNF-α, IL-1β, and iNOS mRNA were significantly increased in the Model group, while the expression levels of Arg-1 and IL-10 mRNA were significantly decreased. Compared to the sh-NC group, the sh-AHNAK group exhibited significantly decreased expression levels of TNF-α, IL-1β, and iNOS mRNA and significantly increased expression levels of Arg-1 and IL-10 mRNA (Fig. 5E to I). Additionally, a critical aspect of macrophage polarization is the change in expression of surface biomarkers. Flow cytometry results showed that compared to the Control group, the proportion of macrophages expressing CD86 was significantly increased in the Model group, while the proportion of macrophages expressing CD206 was significantly decreased. Compared to the sh-NC group, the sh-AHNAK group displayed a significant decrease in the proportion of macrophages expressing

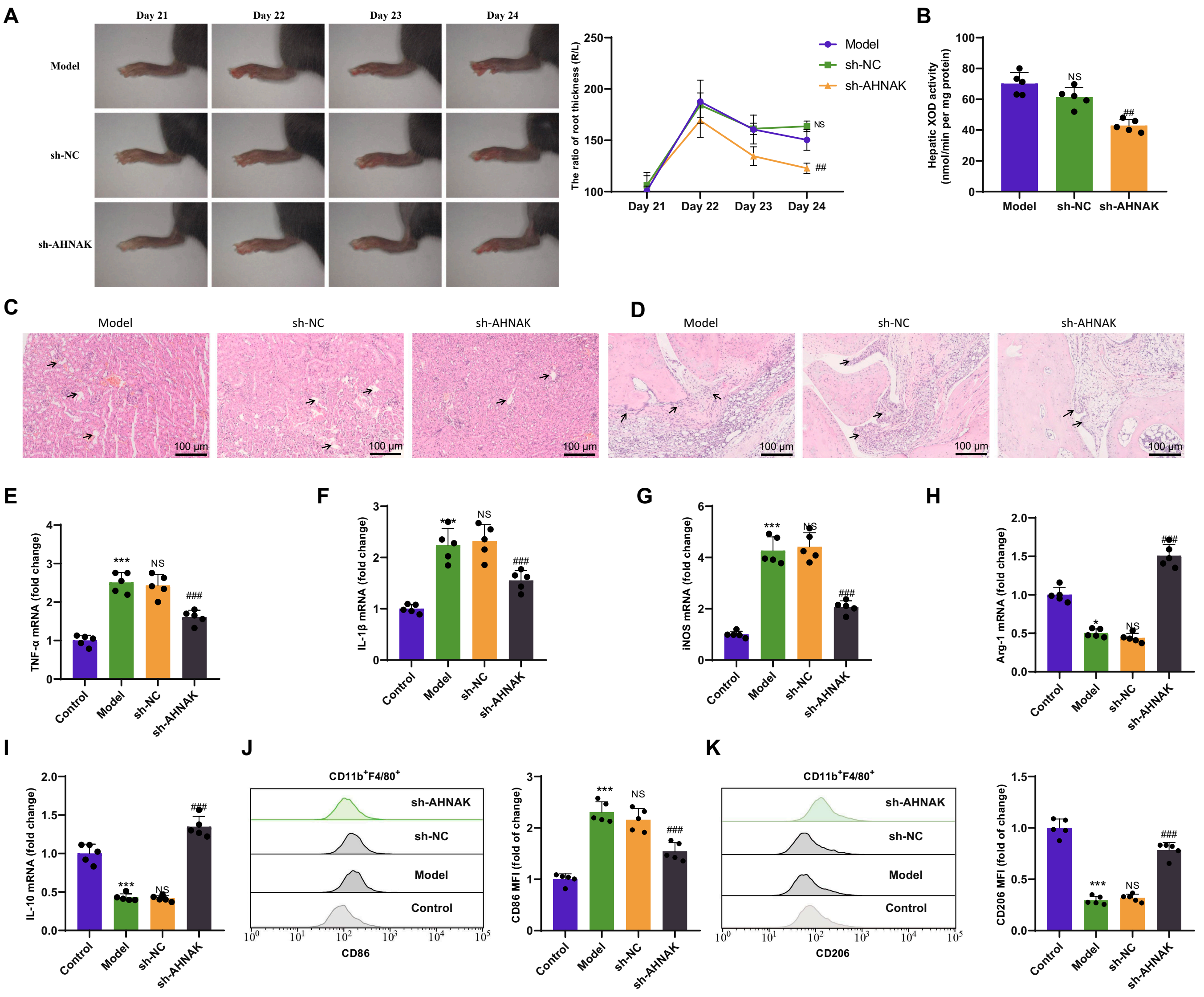

Fig. 5. Investigation of the impact of low AHNAK expression on gout. (A) Representative photos of ankle joints and measurement of ankle swelling ratio. (B) Measurement of XOD activity in mouse livers. (C and D) H&E staining analysis of kidney (C) and ankle joint (D) tissue pathology in different groups of mice, with black arrows indicating cellular vacuolation, synovial hyperplasia, etc. (scale bar, 100 μm). (E to G) RT-qPCR analysis of mRNA expression of M1 macrophage biomarkers TNF-α (E), IL-1β (F), and iNOS (G) in synovial tissue. (H and I) RT-qPCR analysis of mRNA expression of M2 macrophage biomarkers Arg-1 (H) and IL-10 (I) in synovial tissue. (J and K) Flow cytometric analysis of average fluorescence intensity and statistical results of M1 (J) and M2 (K) macrophage populations in synovial tissue of different mouse groups. $P < 0.05$ indicates significant difference compared to the Control group. $P < 0.01$ indicates significant difference compared to the Control group. ** indicates significant difference compared to the Control group at $P < 0.001$. NS indicates no difference compared to the Model group. # indicates significant difference compared to the sh-NC group at $P < 0.05$. ## indicates significant difference compared to the sh-NC group at $P < 0.01$. ### indicates significant difference compared to the sh-NC group at $P < 0.001$. $N = 5$.

CD86 and a significant increase in the proportion of macrophages expressing CD206 (Fig. 5J and K).

Targeted drug delivery of R4F-NM@F127 nanocarriers for the treatment of gout

The synthesis process of R4F-NM@F127 consists of 3 steps (Fig. 6A). First, neutrophils were isolated from mouse peripheral blood with a purity greater than 95%, displaying typical nuclear lobulation morphology after Giemsa staining (Fig. S1C). Subsequently, NMs were obtained through homogenization. Pluronic F127 was then mixed with NMs, and NM@F127 nanocarriers were obtained through a programmed extrusion process. Finally, R4F-NM@F127 was obtained by functionalizing NM@F127 with apoA-I mimetic peptide (R4F), which can bind to phospholipids on NMs, resulting in a core–shell structure of R4F-NM@F127 as observed by TEM after negative staining with phosphotungstic acid (Fig. 6B). The average size, zeta potential, and polydispersity index (PDI) of R4F-NM@F127 were determined using a Malvern Zetasizer Nano ZS analyzer. The results indicated that the particle size of the material was 49.31 ± 3.82 nm (Fig. 6C and D), the zeta potential was −2.76 ± 0.54 mV (Fig. 6E), and the PDI was 0.08 ± 0.005 (Fig. 6F). To investigate the stability of R4F-NM@F127, we continuously monitored the particle size changes of R4F-NM@F127 through dynamic light scattering (DLS) analysis. It was observed that from day 1 to day 3, the particle size of R4F-NM@F127 remained relatively stable over time, while the particle size of NM@F127 increased with time, indicating that R4F-NM@F127 particles are more stable than NM@F127 particles (Fig. 6G). To

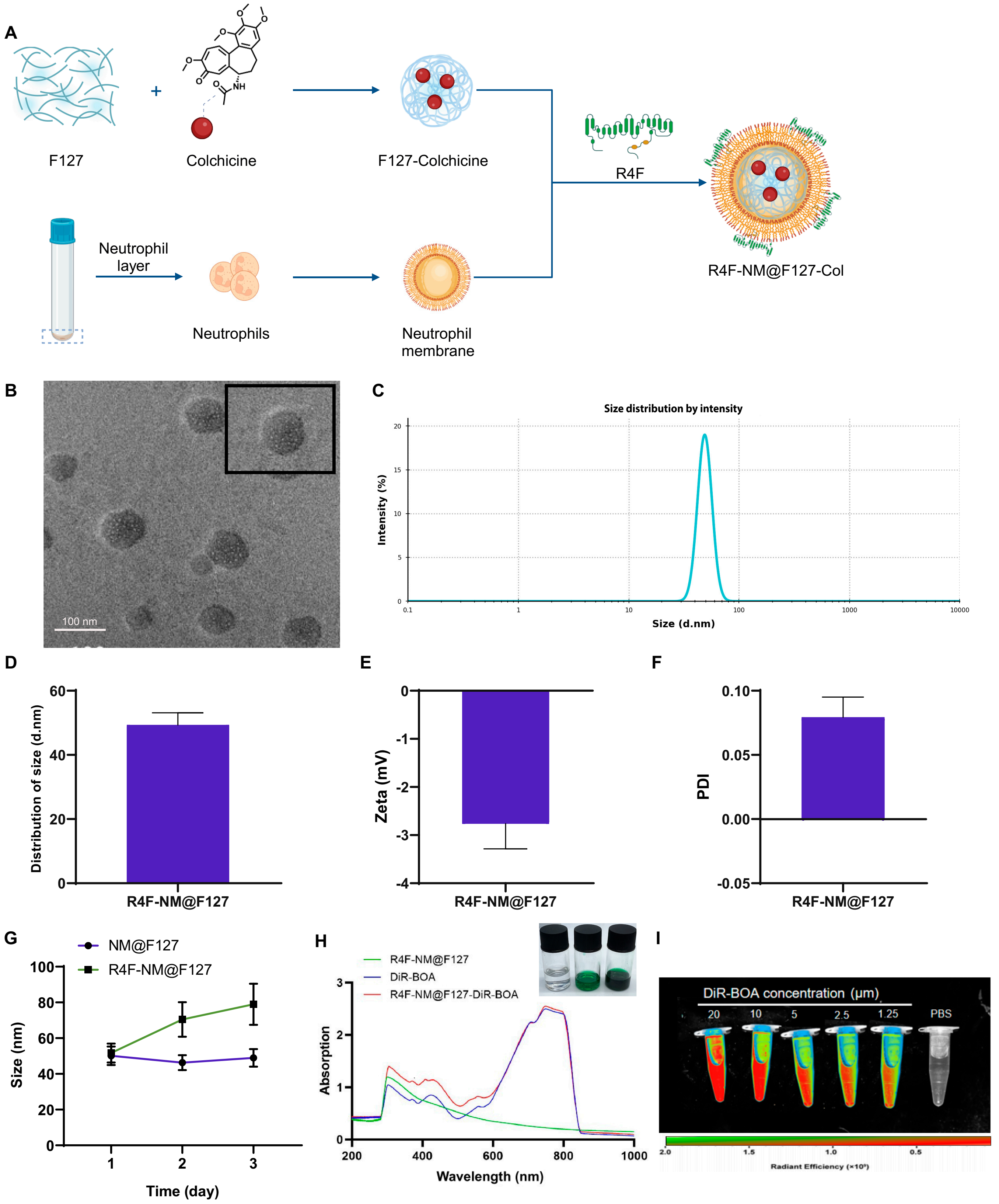

Fig. 6. Preparation and characterization of R4F-NM@F127. (A) Synthetic diagram of biomimetic nanoparticles (R4F-NM@F127) anchored with NM as nanocarriers for colchicine delivery. (B) Representative TEM image of R4F-NM@F127, with enlarged image shown at the top right (scale bar, 100 nm). (C and D) Average particle size of R4F-NM@F127. (E) Zeta potential of R4F-NM@F127. (F) PDI of R4F-NM@F127. (G) Stability of NM@F127 and R4F-NM@F127 monitored by DLS analysis (tested in aqueous solution). (H) Bright-field images and UV-Vis absorption spectra of R4F-NM@F127, free DiR-BOA, and DiR-BOA-labeled R4F-NM@F127. (I) Fluorescence images of DiR-BOA-labeled R4F-NM@F127 at various concentrations. $N = 5$.

evaluate the drug loading capacity of R4F-NM@F127, we used a near-infrared dye (DiR-BOA) as a lipophilic model drug for detection. Visual inspection under white light revealed that R4F-NM@F127 exhibited a distinct dark green color after encapsulating DiR-BOA. Additionally, the UV-Vis absorption spectra demonstrated that both free DiR-BOA and DiR-BOA-labeled R4F-NM@F127 showed enhanced peaks at 730 nm, whereas characteristic absorption peaks were not observed in the absorption spectra of R4F-NM@F127 alone, indicating excellent drug encapsulation performance of R4F-NM@F127 (Fig. 6H). Furthermore, fluorescence imaging data showed that the fluorescence signal of DiR-BOA-labeled R4F-NM@F127 increased with increasing concentrations compared to PBS (Fig. 6I).

Evaluation of the inflammatory joint accumulation and targeting ability of R4F-NM@F127 in gouty mice

To assess the ability of R4F-NM@F127 to remain in circulation for an extended period and preferentially accumulate in inflamed joints, we intravenously injected F127, R4F-NM@F127, and free DiR-BOA-labeled agents into gouty mice. The fluorescence signal of DiR-BOA in the joints was dynamically monitored using an in vivo imaging system at 1, 3, 6, 12, and 24 h after injection. Compared to the free DiR-BOA group, the F127 group showed only weak fluorescence signals in the legs of the gouty mice, indicating the passive accumulation of minimal amounts of DiR-BOA-labeled F127 in the arthritic sites. In contrast, the R4F-NM@F127 group exhibited strong fluorescence signals in the joints of the gouty mice at 1, 3, 6, 12, and 24 h after drug administration, suggesting the accumulation of R4F-NM@F127 in inflammatory joints due to NM coating (Fig. 7A). After imaging, we dissected the mouse organs and joint tissues to further evaluate the arthritis-targeting capability of R4F-NM@F127. Ex vivo imaging also demonstrated (Fig. 7B) intense DiR-BOA fluorescence signals of R4F-NM@F127 in arthritic joints.

After 24 h, we assessed the distribution of R4F-NM@F127 in the serum through ex vivo serum imaging. Imaging data and quantitative analysis of DiR-BOA fluorescence intensity showed a strong fluorescent signal in the serum of the R4F-NM@F127 group (Fig. 7C). These results further confirmed the prolonged circulation time of NM@F127 and R4F-NM@F127 in the bloodstream. Subsequently, we collected synovial fluid to evaluate the distribution of R4F-NM@F127. Clear ex vivo imaging (Fig. 7D) revealed higher DiR-BOA fluorescence signal intensity in the synovial fluid of collagen-induced arthritis (CIA) mice injected with NM@F127 compared to the free DiR-BOA and F127 group.

To further assess the colocalization of R4F-NM@F127 with synovial macrophages, immunofluorescence staining was performed on the synovial membrane. Confocal imaging data displayed (Fig. 7E) weak DiR-BOA fluorescence signals in the synovial membrane of the free DiR-BOA and F127 groups, while the R4F-NM@F127 group exhibited strong signal intensity. Additionally, R4F-NM@F127 labeled with DiR-BOA showed good colocalization with synovial macrophages.

R4F-NM@F127-Col exerts stronger regulatory effects on macrophage polarization

We synthesized the Pluronic F127 polymer carrier loaded with colchicine using a thin-film hydration method, resulting in the formation of R4F-NM@F127-Col. Characterization results indicated that the average particle size and charge of R4F-NM@F127-Col were nearly identical to those of R4F-NM@F127 (Fig. S7A to C). UV-Vis absorption spectra revealed prominent enhancement peaks at 350 nm for both free colchicine and R4F-NM@F127-Col. Additionally, white light images demonstrated a noticeable color change from colorless to yellow for R4F-NM@F127 after encapsulation of colchicine, highlighting a distinct alteration in its appearance (Fig. S7D). The drug loading capacity and in vitro release of R4F-NM@F127-Col were determined using HPLC (Fig. S7E and F). The results indicated a loading efficiency of approximately 78% and a drug loading of about 2%. As shown in Fig. S7F, only around 3% of colchicine was released after 1 h, with approximately 20% remaining unreleased after 72 h, demonstrating the sustained release characteristics of R4F-NM@F127-Col [27]. Furthermore, to assess the in vitro degradation time of R4F-NM@F127-Col more effectively, we evaluated the degradation curve by measuring the weight loss (%) at each time interval. Experimental findings revealed that after 2 weeks of incubation in standard culture media, R4F-NM@F127-Col exhibited a reduction in weight of over 85% from its initial weight (Fig. S7G).

Subsequently, we assessed the cytotoxicity of R4F-NM@F127-Col on RAW264.7 cells using the MTS assay. When the concentration of R4F-NM@F127-Col reached 2 μM (the final colchicine concentration in the culture medium), the cell viability was approximately 98.5% (Fig. 8A). Conversely, at colchicine concentrations of 2 and 4 μM in the culture medium, the cell viabilities for the colchicine group were about 69.3% and 44.1%, respectively, indicating a significant reduction in colchicine-induced cytotoxicity on macrophage cells by the biomimetic nanocarrier system.

Subsequently, we evaluated the ability of R4F-NM@F127-Col to regulate cytokine secretion using an LPS-induced pro-inflammatory model. The mRNA expression levels of key biomarkers for M1 and M2 macrophages were examined by RT-qPCR. The results demonstrated that, compared to normal macrophages (Normal group), LPS stimulation significantly increased the mRNA levels of pro-inflammatory cytokines, which were dose-dependently reduced by colchicine and R4F-NM@F127-Col treatment (Fig. 8B to D). Meanwhile, LPS stimulation markedly decreased the levels of anti-inflammatory cytokines, while colchicine and R4F-NM@F127-Col treatment dose-dependently increased the levels of anti-inflammatory cytokines (Fig. 8E and F). Subsequently, we assessed the impact of R4F-NM@F127-Col on the activation of macrophages in RAW264.7 cells. Following the addition of R4F-NM@F127-Col (colchicine final concentration at 0.5 μM) and LPS to the culture medium, the cells were incubated for 12 h. The results of flow cytometry (Fig. 8G and H) showed that compared to the LPS group, both colchicine and R4F-NM@F127-Col treatment reduced the proportion of macrophages expressing CD86 and increased the proportion of macrophages expressing CD206. Furthermore, Western blot analysis was performed to assess the protein expression levels of iNOS (M1) and Arg-1 (M2) related to macrophage polarization (Fig. 8I to K). It was observed that colchicine and R4F-NM@F127-Col treatment significantly attenuated the elevated expression of iNOS protein induced by LPS stimulation, while the expression of Arg-1 protein, which was reduced by LPS stimulation, was significantly enhanced. The effect of R4F-NM@F127-Col treatment was more pronounced than that of colchicine.

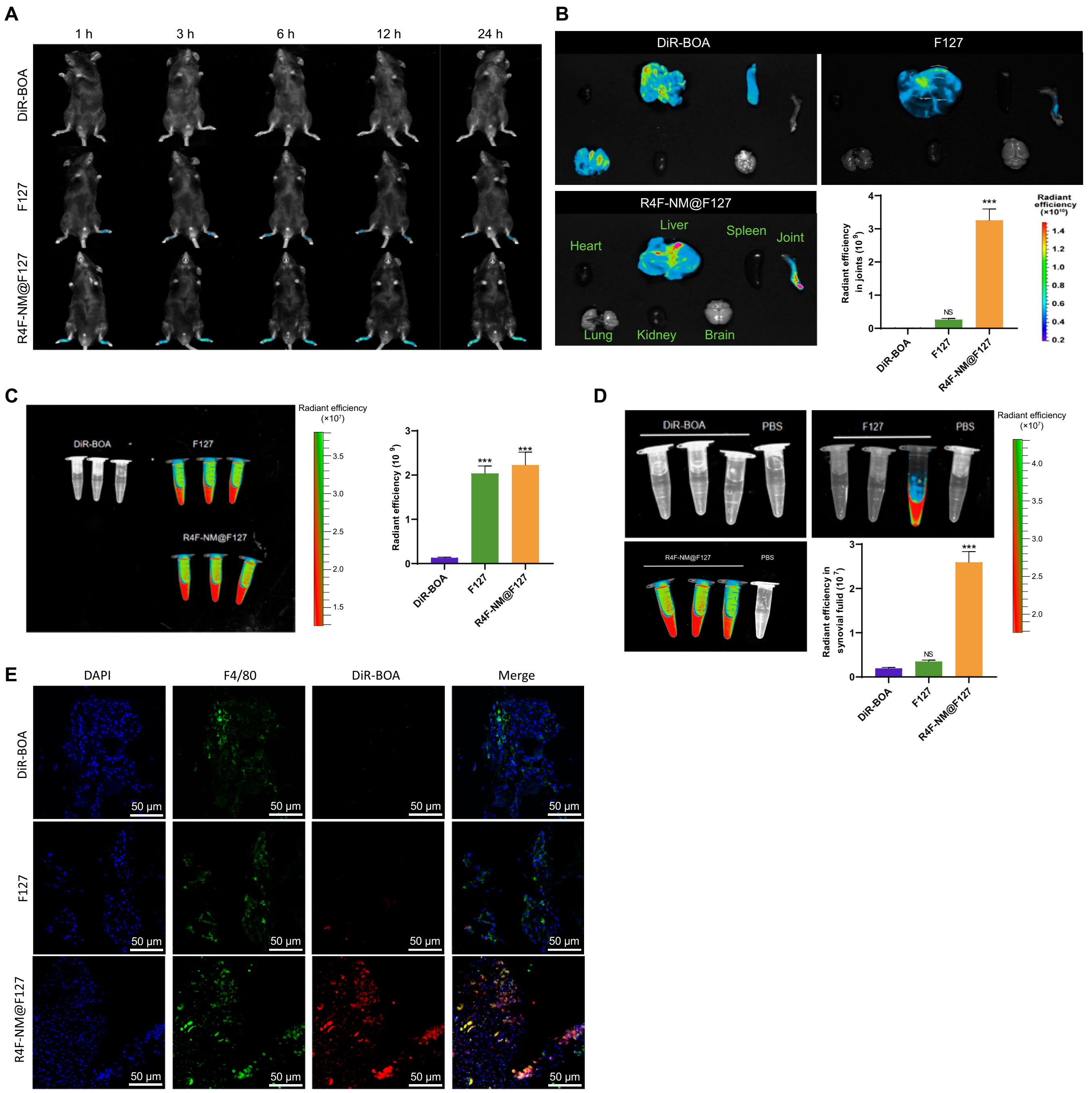

Fig. 7. Distribution of R4F-NM@F127 in gout mice. (A) Real-time fluorescence imaging of gout mice after intravenous injection of DiR-BOA-labeled F127, R4F-NM@F127, and free DiR-BOA. (B) Quantitative analysis of ex vivo organ imaging at 24 h after injection and joint radiance efficiency. (C) Fluorescence imaging of gout mouse serum and quantitative analysis of serum radiance efficiency after injection of DiR-BOA-labeled F127, R4F-NM@F127, and free DiR-BOA. (D) Fluorescence imaging of gout mouse synovial fluid and quantitative analysis of synovial fluid radiance efficiency after injection of DiR-BOA-labeled F127, R4F-NM@F127, and free DiR-BOA. (E) Fluorescence image of cryosectioned synovial tissue (scale bar, 50 nm). *** indicates a significant difference compared to the DiR-BOA group at $P < 0.001$. NS indicates no difference compared to the DiR-BOA group. $N = 3$.

R4F-NM@F127-Col effectively treats gout in mice and modulates macrophage polarization

To further evaluate the therapeutic effect of R4F-NM@F127-Col on gout, we performed intravenous injections of colchicine and R4F-NM@F127-Col in gout mice 24 h after modeling. The degree of joint swelling was quantitatively analyzed using a micrometer caliper, and we observed a significant improvement in joint swelling of the right joints in the colchicine and R4F-NM@F127-Col groups compared to the Model group (Fig. 9A). Furthermore, we measured the levels of AHNAK protein in mouse serum by Western blotting. The results showed a significant up-regulation of AHNAK protein levels in the serum of mice in the Model group compared to the Control group, while both the colchicine and R4F-NM@F127-Col groups

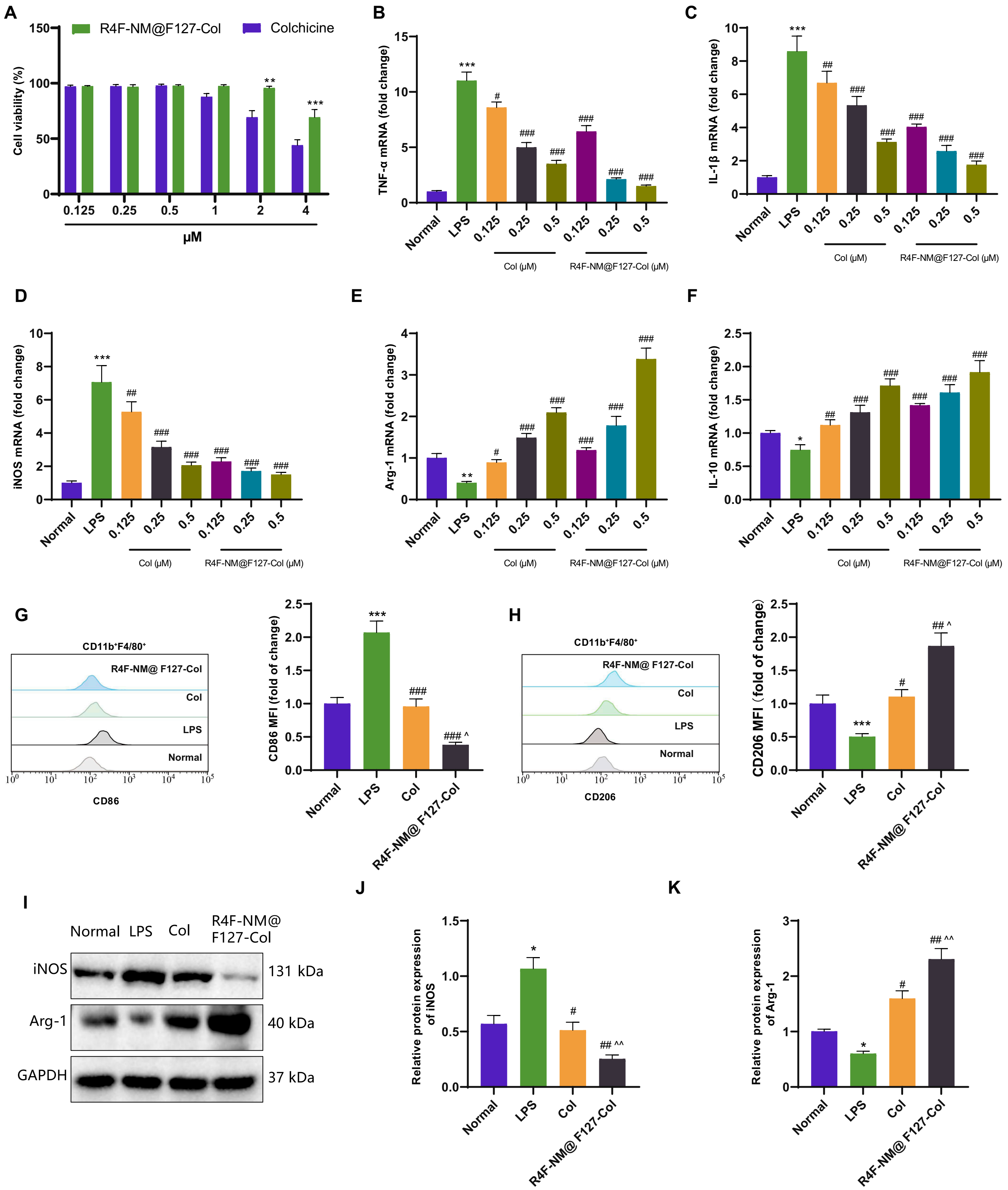

Fig. 8. In vitro study on the effect of R4F-NM@F127-Col on macrophage polarization. (A) Effects of R4F-NM@F127-Col and colchicine on the viability of RAW264.7 cells (x axis represents different final concentrations of colchicine in the culture medium). (B to D) RT-qPCR analysis of TNF-α (B), IL-1β (C), and iNOS (D) mRNA expression, which are biomarkers of M1 macrophages, in RAW264.7 cells. (E and F) RT-qPCR analysis of Arg-1 (E) and IL-10 (F) mRNA expression, which are biomarkers of M2 macrophages, in RAW264.7 cells. (G and H) Flow cytometric analysis of the mean fluorescence intensity of CD86 (G) and CD206 (H) expression in RAW264.7 cells. (I to K) Western blot analysis of iNOS and Arg-1 protein expression in RAW264.7 cells. * indicates $P < 0.05$ compared to the Normal group. ** indicates $P < 0.01$ compared to the Normal group. *** indicates $P < 0.001$ compared to the Normal group. # indicates $P < 0.05$ compared to the LPS group. ## indicates $P < 0.01$ compared to the LPS group. ### indicates $P < 0.001$ compared to the LPS group. ^ indicates $P < 0.05$ compared to the Col group. ^^ indicates $P < 0.01$ compared to the Col group. All cell experiments were repeated 3 times.

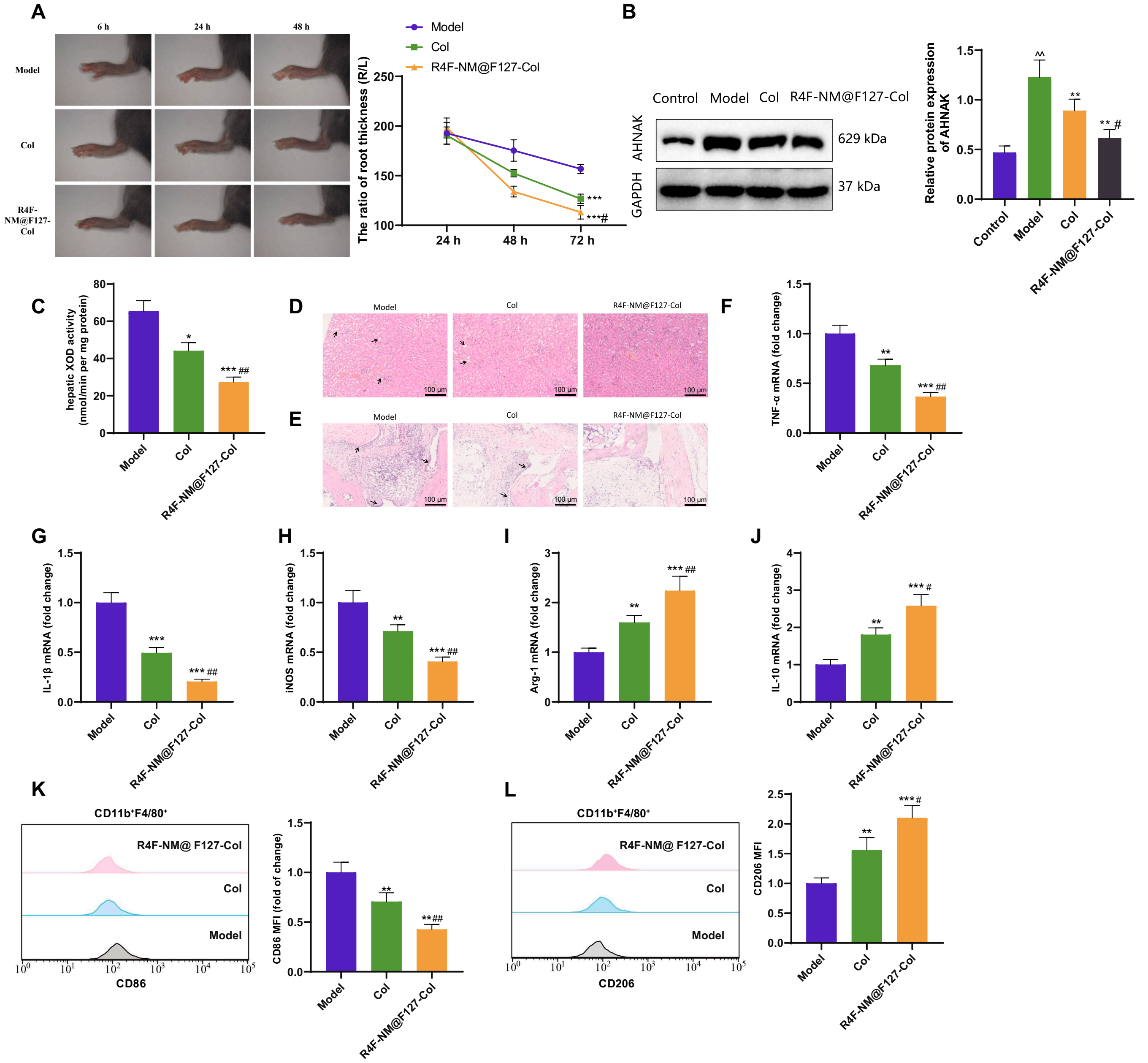

Fig. 9. Investigation of the therapeutic effect of R4F-NM@F127-Col on gout. (A) Representative photographs of ankle joints in each group of mice and the ratio of ankle joint swelling measurement. (B) Western blot analysis of AHNAK protein expression in mouse serum. (C) Measurement of XOD activity in the liver of mice in each group. (D and E) Histopathological analysis of kidney and ankle joint tissues in mice in each group using H&E staining. Black arrows indicate features such as cell vacuolation and synovial hyperplasia (scale bar, 100 μm). (F to H) RT-qPCR analysis of TNF-α (F), IL-1β (G), and iNOS (H) mRNA expression, which are biomarkers of M1 macrophages, in synovial tissues of the joints. (I and J) RT-qPCR analysis of Arg-1 (I) and IL-10 (J) mRNA expression, which are biomarkers of M2 macrophages, in synovial tissues of the joints. (K and L) Flow cytometric analysis of the mean fluorescence intensity and statistical results of M1 and M2 macrophage populations in synovial tissues of the joints. * indicates $P < 0.05$ compared to the Model group. ** indicates $P < 0.01$ compared to the Model group. *** indicates $P < 0.001$ compared to the Model group. # indicates $P < 0.05$ compared to the Col group. ## indicates $P < 0.01$ compared to the Col group. $N = 5$.

exhibited a significant down-regulation of AHNAK protein levels compared to the Model group (Fig. 9B).

We also measured the levels of uric acid, creatinine, and BUN in mouse serum and urine. The results demonstrated that the serum levels of uric acid, creatinine, and BUN were significantly reduced in both the colchicine and R4F-NM@F127-Col groups compared to the Model group, with a more pronounced decrease in the R4F-NM@F127-Col group. Conversely, the urine levels of uric acid, creatinine, and the FEUA value were significantly increased, with a higher increase in the R4F-NM@F127-Col group (Table S3). Furthermore, the liver XOD activity was significantly reduced in both the colchicine and R4F-NM@F127-Col groups compared to the Model group. In comparison to the colchicine group, the R4F-NM@F127-Col group exhibited even lower liver XOD activity (Fig. 9C). Histological staining of H&E (Fig. 9D and E) showed improvements in renal tubular interstitial and knee joint inflammation, as well as alleviation of synoviocyte necrosis in the colchicine and R4F-NM@F127-Col groups.

We assessed the inhibitory effect of R4F-NM@F127-Col on AHNAK-induced macrophage polarization in vivo by evaluating the expression of M1 and M2 macrophage-specific phenotypic markers in mouse synovial tissue. RT-qPCR was employed to detect the mRNA expression levels of key biomarkers, including TNF-α, IL-1β, and iNOS for M1 macrophages, as well as Arg-1 and IL-10 for M2 macrophages. The results (Fig. 9F to J) demonstrated that the mRNA expression levels of TNF-α, IL-1β, and iNOS were significantly decreased in both the colchicine and R4F-NM@F127-Col groups compared to the Model group, while the expression levels of Arg-1 and IL-10 were significantly increased. Flow cytometry results (Fig. 9K and L) showed a significant decrease in the proportion of CD86-expressing macrophages and an increase in the proportion of CD206-expressing macrophages in both the colchicine and R4F-NM@F127-Col groups compared to the Model group. The R4F-NM@F127-Col group outperformed the colchicine group in all measured indicators.

Good biocompatibility of R4F-NM@F127-Col nanoparticles in vivo

One critical concern of nanoparticle therapy is their potential toxicity to normal tissues. Therefore, a toxicity evaluation of the nanoparticles was conducted on the 14th day after treatment. The results revealed that the color and size of the heart, spleen, lungs, kidneys, and brain in all groups of mice were normal. Furthermore, histological examination of these organs showed normal physiological structure and cellular morphology. In contrast, the colchicine treatment group exhibited histological liver damage, including inflammation cell infiltration, hepatocyte necrosis, and nuclear fragmentation (Fig. S8A). Additionally, biochemical markers of liver damage, ALT, and AST activity were significantly elevated (Fig. S8B and C).

Discussion

Gout is an inflammatory disease caused by disrupted uric acid metabolism. However, current treatments for gout still have certain limitations [31,32]. Colchicine is a frontline and traditional drug for treating acute gouty arthritis. Its main mechanism involves suppressing leukocyte activity to reduce inflammation and alleviate symptoms, although the specific anti-inflammatory targets remain unclear. However, colchicine use often accompanies severe side effects, including substantial gastrointestinal toxicity, impairing liver metabolism and enterohepatic circulation, slowing drug metabolism, and exhibiting dose-dependent toxicity [33]. To mitigate the side effects associated with gout treatment, we attempted to utilize natural cells as drug delivery carriers, specifically cell membrane biomimetic nanodrugs, to minimize harm to the human body. Neutrophils are the most abundant cell type in synovial fluid, with a notable increase in IL-8, indicating their natural role as carriers for targeted drug delivery in synovial fluid [26]. In recent years, researchers have developed biomimetic drug delivery systems with inflammatory targeting functions using NMs containing key molecules [26,34]. In this study, we employed peptide-anchored NM-coated biomimetic nanoparticles (R4F-NM@F127) as a nanocarrier for colchicine delivery, aiming for targeted drug transport in gout. The purpose of this study is to explore the effect and mechanism of colchicine carried by R4F-NM@F127 nanoparticles on improving gout inflammation and achieving modulation of macrophage polarization. The substantial of this research objective lies in the need for a deeper understanding of the pathophysiology of gout and the search for novel treatment strategies capable of effectively regulating macrophage polarization.

Using scRNA-seq technology, we have revealed the critical role of macrophage polarization in gout [35,36]. Compared to previous studies, we have identified more genes related to macrophage polarization, which further deepens our understanding of the development of gout inflammation and provides a basis for the development of new treatment strategies [37,38].

AHNAK is a huge protein (700 kDa) initially discovered in human neuroblastoma and skin epithelial cells, responsible for regulating cytoskeletal formation, muscle regeneration, calcium homeostasis, and signaling transduction. Studies have identified AHNAK as a novel tumor suppressor that inhibits M2 alternative activation of tumor-promoting macrophages [39]. Colchicine's binding to AHNAK protein has been confirmed through molecular docking and SPR experiments [40]. Consistent with the results of other related studies, this finding further supports the interaction between colchicine and AHNAK protein, demonstrating the reliability and feasibility of colchicine's action [41,42]. The identification of AHNAK protein as a key target of colchicine provides a clue for further investigation into the mechanism of its action.

By employing lentiviral intervention, we attenuated the expression level of AHNAK in mice and observed a series of associated symptomatic improvements. The intervention with colchicine and R4F-NM@F127-Col effectively reduced AHNAK expression levels in mice, modulated macrophage polarization, and ameliorated symptoms in gout-afflicted mice [13,43]. This experiment further validates the effectiveness of colchicine as a potential drug for treating gout.

Nanoparticle accumulation was observed at high levels in the liver (Fig. 7B). The liver serves as a primary site for drug metabolism; upon nanoparticle arrival, it may be internalized by hepatocytes for metabolic processing. During this process, drugs may undergo breakdown into smaller molecules or transform into more easily excretable forms through conjugation reactions. Subsequently, they are eliminated via bile into the intestine and eventually expelled through feces (PMCID: PMC7508170). Hence, the liver can exhibit a certain degree of nanoparticle aggregation. Given the liver's role as a pivotal site for drug metabolism, drug intake generally carries inherent hepatotoxicity risks. Prior studies have extensively documented information on apoA-I mimetic peptide 4F, with initial clinical assessments in high-risk computer-aided design (CAD) patients using D-4F, indicating that subjects tolerated single oral doses of D-4F (30, 100, 300, or 500 mg) safely and with good tolerability [44]. Hence, appropriate drug dosages can mitigate adverse effects on other organs.

In summary, we can draw the following preliminary conclusions: Colchicine inhibits the polarization of macrophages toward the pro-inflammatory M1 phenotype by binding to AHNAK protein, inducing the polarization of the anti-inflammatory M2 phenotype and subsequently alleviating gout. R4F-NM@F127-loaded colchicine can serve as a targeted therapeutic drug to modulate M1/M2 macrophage polarization, alleviating gout while reducing toxicity to normal tissue (Fig. 10).

The scientific value of this study lies in exploring the mechanisms by which colchicine, encapsulated in R4F-NM@F127 nanoparticles, improves inflammation in gout. Through this research, we have revealed the significant role of macrophage polarization in gout and identified core regulatory genes that

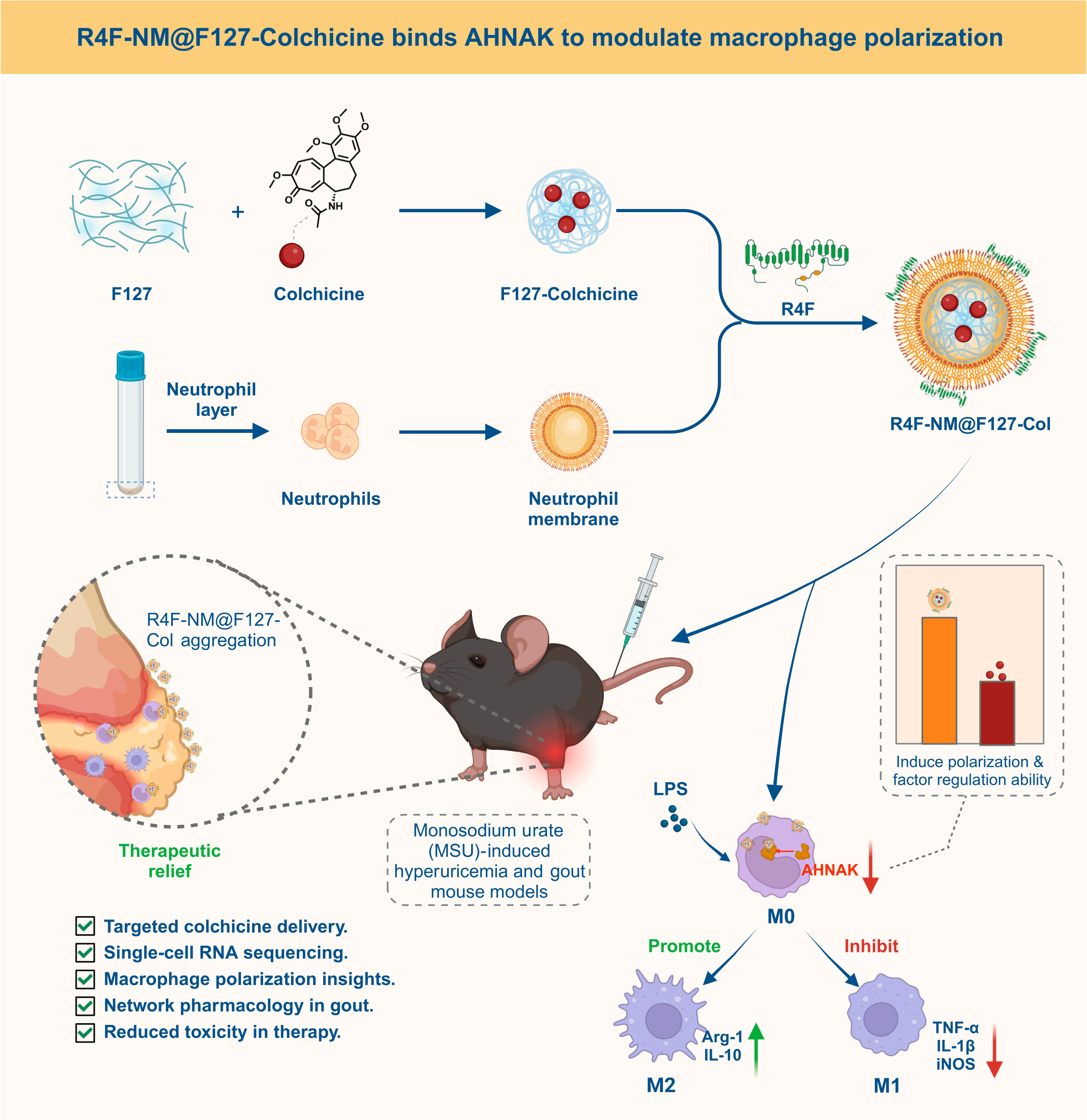

Fig. 10. R4F-NM@F127 loaded with colchicine improves gout by modulating macrophage polarization through binding to AHNAK protein.

affect macrophage polarization. In addition, we have verified the binding of colchicine and AHNAK protein and confirmed that the improvement of relevant indicators and macrophage polarization state in gout mice can be achieved by modulating this key gene. This study helps to deepen our understanding of the pathogenesis of gout inflammation and provides new drug targets for gout treatment.

The clinical value of this study lies in the use of R4F-NM@F127 nanoparticles as a targeted therapeutic drug to regulate M1/M2 macrophage polarization, thereby alleviating gout symptoms and reducing toxicity to normal tissue. This treatment strategy has potential clinical application value and can provide more effective and safer treatment options for gout patients. At the same time, this study also provides new ideas and methods for the development of other drugs that target macrophage polarization.

However, this study has several limitations. First, we utilized a mouse model for study convenience, thereby limiting the direct applicability of our findings to human clinical practice. Second, while we validated the therapeutic effects of colchicine in both in vitro and in vivo studies, further clinical research is necessary to confirm its safety and efficacy. Additionally,

although our study indicated that colchicine intervention can suppress AHNAK expression in mice and molecular docking and SPR analysis demonstrated a high-affinity interaction between colchicine and AHNAK protein, the specific mechanisms of action remain unverified and warrant further exploration. Lastly, the focus of this study was on the regulation of macrophage polarization by inhibiting AHNAK, yet the specific mechanisms underlying AHNAK regulation of macrophage polarization and other potential pathways in gout inflammation remain inadequately explored.

Looking forward, future research can further delve into the molecular mechanisms of macrophage polarization regulation and the impact of colchicine's binding to AHNAK protein. Additionally, the preparation methods and characteristics of R4F-NM@F127 nanoparticles can be further optimized to enhance drug loading and targeting, thereby improving treatment effectiveness. Further clinical studies can be conducted to verify the safety and efficacy of colchicine and R4F-NM@F127 nanoparticles, as well as explore their prospects in clinical practice.

Ethical Approval

All animal experiments were approved by the Animal Ethics Committee of Shengjing Hospital Affiliated to China Medical University.

Acknowledgments

Funding: This work was supported by the National Natural Science Fund (No: 81241083), the Science and Technology Department Project of Liaoning Province (Nos: 2021JH2/10300087), and the Science Plan Program of Shenyang City (Nos: 21-172-9-08).

Author contributions: N.Z. and L.Z. conceived and designed the study. J.L. and H.L. conducted the experiments and analyzed the data. Y.C. supervised the project and contributed to the interpretation of the results. All authors contributed to drafting the manuscript and approved the final version for publication.

Competing interests: The authors declare that they have no competing interests.

Data Availability

All data can be provided as needed.

Supplementary Materials

Figs. S1 to S8
Tables S1 to S7

References

1. Dalbeth N, Gosling AL, Gaffo A, Abhishek A. Gout. *Lancet*. 2021;397(10287):1843–1855.
2. Giannopoulos G, Angelidis C, Deftereos S. Gout and arrhythmias: In search for causation beyond association. *Trends Cardiovasc Med*. 2019;29(1):41–47.
3. He Y, Xue X, Terkeltaub R, Dalbeth N, Merriman TR, Mount DB, Feng Z, Li X, Cui L, Liu Z, et al. Association of acidic urine pH with impaired renal function in primary gout patients: A Chinese population-based cross-sectional study. *Arthritis Res Ther*. 2022;24(1):32.
4. Dalbeth N, Horne A, Mihov B, Stewart A, Gamble GD, Merriman TR, Stamp LK, Reid IR. Elevated urate levels do not alter bone turnover markers: Randomized controlled trial of inosine supplementation in postmenopausal women. *Arthritis Rheumatol*. 2021;73(9):1758–1764.
5. Singh JA, Edwards NL. Gout management and outcomes during the COVID-19 pandemic: A cross-sectional internet survey. *Ther Adv Musculoskelet Dis*. 2020;12:1759720X20966124.
6. Gelber AC. Treatment guidelines in gout. *Rheum Dis Clin N Am*. 2022;48(3):659–678.
7. Peace CG, O'Neill LA. The role of itaconate in host defense and inflammation. *J Clin Invest*. 2022;132(2):Article e148548.
8. Lan Z, Chen L, Feng J, Xie Z, Liu Z, Wang F, Liu P, Yue X, du L, Zhao Y, et al. Mechanosensitive TRPV4 is required for crystal-induced inflammation. *Ann Rheum Dis*. 2021;80(12):1604–1614.
9. Li J, Jiang X, Li H, Gelinsky M, Gu Z. Tailoring materials for modulation of macrophage fate. *Adv Mater*. 2021;33(12):Article e2004172.
10. Ballini A, Dipalma G, Isacco CG, Boccellino M, di Domenico M, Santacroce L, Nguyễn KCD, Scacco S, Calvani M, Boddi A, et al. Oral microbiota and immune system crosstalk: A translational research. *Biology*. 2020;9(6):131.
11. Zhou J, Sun S, He Y, Yan T, Sun J, Pan J, Zhu S, Chen L, Zhu P, Xu B, et al. Role of magnesium-doped calcium sulfate and β-tricalcium phosphate composite ceramics in macrophage polarization and osteo-induction. *Odontology*. 2022;110(4):735–746.
12. Zhu J, Li C, Wang P, Liu Y, Li Z, Chen Z, Zhang Y, Wang B, Li X, Yan Z, et al. Deficiency of salt-inducible kinase 2 (SIK2) promotes immune injury by inhibiting the maturation of lymphocytes. *MedComm*. 2023;4(5):Article e366.
13. Zhang FS, He QZ, Qin CH, Little PJ, Weng JP, Xu SW. Therapeutic potential of colchicine in cardiovascular medicine: A pharmacological review. *Acta Pharmacol Sin*. 2022;43(9):2173–2190.
14. Deftereos SG, Beerkens FJ, Shah B, Giannopoulos G, Vrachatis DA, Giotaki SG, Siasos G, Nicolas J, Arnott C, Patel S, et al. Colchicine in cardiovascular disease: In-depth review. *Circulation*. 2022;145(1):61–78.
15. Soy M, Keser G, Atagündüz P, Tabak F, Atagündüz I, Kayhan S. Cytokine storm in COVID-19: Pathogenesis and overview of anti-inflammatory agents used in treatment. *Clin Rheumatol*. 2020;39(7):2085–2094.
16. Maranda G, Gagnon D. Gunshot wounds: Evaluation and treatment. *J Dent Que*. 1987;24:337–340.
17. Yan YP, Sailor KA, Lang BT, Park SW, Vemuganti R, Dempsey RJ. Monocyte chemoattractant protein-1 plays a critical role in neuroblast migration after focal cerebral ischemia. *J Cereb Blood Flow Metab*. 2007;27(6):1213–1224.
18. Liang G, Nie Y, Chang Y, Zeng S, Liang C, Zheng X, Xiao D, Zhan S, Zheng Q. Protective effects of Rhizoma smilacis glabrae extracts on potassium oxonate- and monosodium urate-induced hyperuricemia and gout in mice. *Phytomedicine*. 2019;59:Article 152772.
19. Xu S, Zhu W, Shao M, Zhang F, Guo J, Xu H, Jiang J, Ma X, Xia X, Zhi X, et al. Ecto-5'-nucleotidase (CD73) attenuates inflammation after spinal cord injury by promoting macrophages/microglia M2 polarization in mice. *J Neuroinflammation*. 2018;15(1):155.

20. Pan X, Wang J, Guo L, Na F, du J, Chen X, Zhong A, Zhao L, Zhang L, Zhang M, et al. Identifying a confused cell identity for esophageal squamous cell carcinoma. *Signal Transduct Target Ther*. 2022;7(1):122.
21. Butler A, Hoffman P, Smibert P, Papalexi E, Satija R. Integrating single-cell transcriptomic data across different conditions, technologies, and species. *Nat Biotechnol*. 2018;36(5):411–420.
22. Li X, Wei S, Niu S, Ma X, Li H, Jing M, Zhao Y. Network pharmacology prediction and molecular docking-based strategy to explore the potential mechanism of Huanglian Jiedu Decoction against sepsis. *Comput Biol Med*. 2022;144: Article 105389.
23. Bai T, Guo J, Deng Y, Zheng Y, Shang J, Zheng P, Liu M, Yang M, Zhang J. A systematical strategy for quality markers screening of different methods processing Platycodonis radix based on phytochemical analysis and the impact on chronic obstructive pulmonary disease. *J Ethnopharmacol*. 2024;319(Pt 2):Article 117311.
24. Pan W, Li W, Wu H, Xie X, Xie M, Nie Q, Liu Z, Cai S. Aging-accelerated mouse prone 8 (SAMP8) mice experiment and network pharmacological analysis of aged Liupao tea aqueous extract in delaying the decline changes of the body. *Antioxidants*. 2023;12(3):685.
25. Tang Y, Wang X, Li Z, He Z, Yang X, Cheng X, Peng Y, Xue Q, Bai Y, Zhang R, et al. Heparin prevents caspase-11-dependent septic lethality independent of anticoagulant properties. *Immunity*. 2021;54(3):454–467.e6.
26. Yang N, Li M, Wu L, Song Y, Yu S, Wan Y, Cheng W, Yang B, Mou X, Yu H, et al. Peptide-anchored neutrophil membrane-coated biomimetic nanodrug for targeted treatment of rheumatoid arthritis. *J Nanobiotechnology*. 2023;21(1):13.
27. Zheng J, Yang N, Wan Y, Cheng W, Zhang G, Yu S, Yang B, Liu X, Chen X, Ding X, et al. Celastrol-loaded biomimetic nanodrug ameliorates APAP-induced liver injury through modulating macrophage polarization. *J Mol Med*. 2023;101(6):699–716.
28. Zhao K, Zhang Y, Kang L, Song Y, Wang K, Li S, Wu X, Hua W, Shao Z, Yang S, et al. Methylation of microRNA-129-5P modulates nucleus pulposus cell autophagy by targeting Beclin-1 in intervertebral disc degeneration. *Oncotarget*. 2017;8(49):86264–86276.
29. Ban Z, He J, Tang Z, Zhang L, Xu Z. LRG-1 enhances the migration of thyroid carcinoma cells through promotion of the epithelial-mesenchymal transition by activating MAPK/p38 signaling. *Oncol Rep*. 2019;41(6):3270–3280.
30. Leyton-Jaimes MF, Kahn J, Israelson A. Macrophage migration inhibitory factor: A multifaceted cytokine implicated in multiple neurological diseases. *Exp Neurol*. 2018;301(Pt B): 83–91.
31. Shan B, Chen T, Huang B, Liu Y, Chen J. Untargeted metabolomics reveal the therapeutic effects of Ermiao wan categorized formulas on rats with hyperuricemia. *J Ethnopharmacol*. 2021;281:Article 114545.
32. Shimizu M, Naito R, Sato A, Ishiwata S, Yatsu S, Shitara J, Matsumoto H, Murata A, Kato T, Suda S, et al. Diurnal variations in serum uric acid, xanthine, and xanthine oxidoreductase activity in male patients with coronary artery disease. *Nutrients*. 2023;15(20):4480.
33. Bhat A, Naguwa SM, Cheema GS, Gershwin ME. Colchicine revisited. *Ann N Y Acad Sci*. 2009;1173:766–773.
34. Wang D, Wang S, Zhou Z, Bai D, Zhang Q, Ai X, Gao W, Zhang L. White blood cell membrane-coated nanoparticles: Recent development and medical applications. *Adv Healthc Mater*. 2022;11(7):Article e2101349.
35. Wu K, Lin K, Li X, Yuan X, Xu P, Ni P, Xu D. Redefining tumor-associated macrophage subpopulations and functions in the tumor microenvironment. *Front Immunol*. 2020;11:1731.
36. Feng X, Chen W, Ni X, Little PJ, Xu S, Tang L, Weng J. Metformin, macrophage dysfunction and atherosclerosis. *Front Immunol*. 2021;12:Article 682853.
37. Orecchioni M, Ghosheh Y, Pramod AB, Ley K. Macrophage polarization: Different gene signatures in M1(LPS+) vs. classically and M2(LPS-) vs. alternatively activated macrophages. *Front Immunol*. 2019;10:1084.
38. Kishore A, Petrek M. Roles of macrophage polarization and macrophage-derived miRNAs in pulmonary fibrosis. *Front Immunol*. 2021;12:Article 678457.
39. Park JW, Kim IY, Choi JW, Lim HJ, Shin JH, Kim YN, Lee SH, Son Y, Sohn M, Woo JK, et al. AHNAK loss in mice promotes type II pneumocyte hyperplasia and lung tumor development. *Mol Cancer Res*. 2018;16(8):1287–1298.
40. Kode J, Kovvuri J, Nagaraju B, Jadhav S, Barkume M, Sen S, Kasinathan NK, Chaudhari P, Mohanty BS, Gour J, et al. Synthesis, biological evaluation, and molecular docking analysis of phenstatin based indole linked chalcones as anticancer agents and tubulin polymerization inhibitors. *Bioorg Chem*. 2020;105:Article 104447.
41. Angelidis C, Kotsialou Z, Kossyvakis C, Vrettou AR, Zacharoulis A, Kolokathis F, Kekeris V, Giannopoulos G. Colchicine pharmacokinetics and mechanism of action. *Curr Pharm Des*. 2018;24(6):659–663.
42. Leung YY, Yao Hui LL, Kraus VB. Colchicine—Update on mechanisms of action and therapeutic uses. *Semin Arthritis Rheum*. 2015;45(3):341–350.
43. Fine N, Gracey E, Dimitriou I, La Rose J, Glogauer M, Rottapel R. GEF-H1 is required for colchicine inhibition of neutrophil rolling and recruitment in mouse models of gout. *J Immunol*. 2020;205(12):3300–3310.
44. Bloedon LT, Dunbar R, Duffy D, Pinell-Salles P, Norris R, DeGroot BJ, Movva R, Navab M, Fogelman AM, Rader DJ. Safety, pharmacokinetics, and pharmacodynamics of oral apoA-I mimetic peptide D-4F in high-risk cardiovascular patients. *J Lipid Res*. 2008;49(6):1344–1352.
