# Supplementary material for: Harnessing Nanotechnology for Gout Therapy: Colchicine-Loaded Nanoparticles Regulate Macrophage Polarization and Reduce Inflammation
Source: Biomater Res. 2024 Dec 11;28:0089. doi: 10.34133/bmr.0089 (PMC11632155; doi:10.34133/bmr.0089)
Supplement: Supplementary 1 — Figs. S1 to S8 Tables S1 to S7 [file bmr.0089.f1.zip › Supplementary Information.docx]

**Figure S1. Isolation and identification of neutrophils.**

Note: (A) RT-qPCR analysis of the silencing efficiency of three AHNAK shRNAs; (B) Schematic diagram of neutrophil isolation from mouse peripheral blood; (C) Identification of neutrophils using Giemsa staining (left, scale bar: 100 μm); * indicates *P* < 0.05 compared to the Control group; ** indicates *P* < 0.01 compared to the Control group; N = 5.

**Figure S2. Quality control of scRNA-seq data.**

Note: (A) Violin plots of the number of genes (nFeature_RNA), mRNA molecules (nCount_RNA), and percentage of mitochondrial genes (percent.mt) per cell before quality control for the Control and Model groups; (B) Violin plots of the number of genes (nFeature_RNA), mRNA molecules (nCount_RNA), and percentage of mitochondrial genes (percent.mt) per cell after quality control for the Control and Model groups; (C) Scatter plots showing the correlation between nCount_RNA and percent.mt and between nCount_RNA and nFeature_RNA in the post-quality control data; (D) Red represents the top 4000 highly variable genes selected by variance analysis, while black represents low variable genes.

**Figure S3. PCA of scRNA-seq data.**

Note: (A) Distribution of cells on PC_1 and PC_2 before batch correction, where each point represents a cell; (B) Distribution of cells on PC_1 and PC_2 after batch correction, where each point represents a cell; (C-D) Heatmaps showing the expression levels of top genes in the first four PCs before batch correction (yellow denotes upregulation, purple denotes downregulation); (E) Scree plot displaying the variance contribution of each PC; (F) p-values of the top 20 PCs obtained from PCA analysis.

**Figure S4. Clustering and differential analysis of scRNA-seq data.**

Note: (A) t-SNE clustering analysis grouped cells into 15 cell clusters; (B) Heatmaps showing the top 10 marker genes expressed in each cell cluster; (C) Analysis of the proportion differences between cells in the Control and Model groups.

**Figure S5. Differential analysis of ligand-receptor interactions between cells.**

Note: The red box highlights important ligand-receptor pathways in which myeloid cells interact with other immune cells.

**Figure S6. PCA of myeloid cell scRNA-seq data.**

Note: (A-B) Heatmaps illustrating the expression levels of top genes in the first four PCs obtained from PCA analysis (yellow denotes upregulation, purple denotes downregulation); (C) Scree plot displaying the variance contribution of the top 20 PCs obtained from PCA analysis; (D) The scree plot generated by the variance contribution rate of each principal component is shown; (E) t-SNE clustering analysis grouped myeloid cells into 6 cell clusters; (F) Heatmaps showing the expression of the top 10 marker genes in each cell cluster; (G) t-SNE clustering analysis grouped myeloid cells in the Control and Model groups.

**Figure S7. Characterization of R4F-NM@F127-Col.**

Note: (A-B) Average particle size of R4F-NM@F127-Col; (C) Zeta potential of R4F-NM@F127-Col; (D) Bright field images and UV-visible absorption spectra of R4F-NM@F127, Col, and R4F-NM@F127-Col; N=5; (E) Colchicine concentration was analyzed by high-performance liquid chromatography to calculate drug loading and drug release rate; (F) In vitro release curve of colchicine; (G) Degradation curve of R4F-NM@F127-Col in PBS (pH 7.4) at 37°C.

**Figure S8. Toxicity evaluation of R4F-NM@F127-Col nanoparticles.**

Note: (A) HE staining of heart, liver, spleen, lung, kidney, and brain tissues from each group of mice (scale bar: 100 μm); (B-C) Serum levels of ALT (B) and AST (C) in each group of mice; * indicates significant difference compared to the Control group, P < 0.05; N=5.
