# Supplementary material for: Harnessing Nanotechnology for Gout Therapy: Colchicine-Loaded Nanoparticles Regulate Macrophage Polarization and Reduce Inflammation
Source: Biomater Res. 2024 Dec 11;28:0089. doi: 10.34133/bmr.0089 (PMC11632155; doi:10.34133/bmr.0089)
Supplement: Supplementary 1 — Figs. S1 to S8 Tables S1 to S7 [file bmr.0089.f1.zip › Table S1.docx]

**Table S1. RT-qPCR primer sequences (Mouse)**

|  | **Forward Primer (**5′-3′**)** |
| --- | --- |
| iNOS | F: 5′-AATCTTGGAGCGAGTTGTGG-3′  R: 5′-CAGGAAGTAGGTGAGGGCTTG-3′ |
| TNF-α | F: 5′-CCCTCACACTCAGATCATCTTCT-3′  R: 5′-GCTACGACGTGGGCTACAG-3′ |
| IL-1β | F: 5′-TGGACCTTCCAGGATGAGGACA-3′  R: 5′-GTTCATCTCGGAGCCTGTAGTG-3′ |
| Arg-1 | F: 5′-CTCCAAGCCAAAGTCCTTAGAG-3′  R: 5′-AGGAGCTGTCATTAGGGACATC-3′ |
| IL-10 | F: 5′-GCTCCTAGAGCTGCGGACT-3′  R: 5′-TGTTGTCCAGCTGGTCCTTT-3′ |
| IL-6 | F: 5′-TTCCTCTGGTCTTCTGGAGT-3′  R: 5′-TCTGTGACTCCAGCTTATCTCTTG-3′ |
| IL-12 | F: 5′-CTCTTTTTGGCCACCCTTGC-3′  R: 5′-TCTTCAGCAGGTTTCGGGAC-3′ |
| GAPDH | F: 5′-GGAGAGTGTTTCCTCGTCCC-3′  R: 5′-ATGAAGGGGTCGTTGATGGC-3′ |

F: Forward; R: Reverse
