# Supplementary material for: Harnessing Nanotechnology for Gout Therapy: Colchicine-Loaded Nanoparticles Regulate Macrophage Polarization and Reduce Inflammation
Source: Biomater Res. 2024 Dec 11;28:0089. doi: 10.34133/bmr.0089 (PMC11632155; doi:10.34133/bmr.0089)
Supplement: Supplementary 1 — Figs. S1 to S8 Tables S1 to S7 [file bmr.0089.f1.zip › Table S2.docx]

**Table S2. Western blot test of primary antibody manufacturer information**

| Antibody name | Antibody item number | concentration | manufacturer |
| --- | --- | --- | --- |
| Anti-iNOS | ab178945 | 1: 1000 | Abcam |
| Anti-Arg-1 | 93668 | 1: 1000 | CST |
| Anti-AHNAK | ab68556 | 1: 1000 | Abcam |
