# Supplementary material for: Harnessing Nanotechnology for Gout Therapy: Colchicine-Loaded Nanoparticles Regulate Macrophage Polarization and Reduce Inflammation
Source: Biomater Res. 2024 Dec 11;28:0089. doi: 10.34133/bmr.0089 (PMC11632155; doi:10.34133/bmr.0089)
Supplement: Supplementary 1 — Figs. S1 to S8 Tables S1 to S7 [file bmr.0089.f1.zip › Table S3.docx]

**Table S3. The indicators of PO- and MSU-induced hyperuricemic and gouty mice**

| Group | Urine, mg/dl | | Serum, mg/dl | | BUN, mg/dl | FEUA |
| --- | --- | --- | --- | --- | --- | --- |
|  | Uric acid | Creatinine | Uric acid | Creatinine |  |  |
| Control | 102.4 ± 4.2 | 105.6 ± 5.3 | 4.5 ± 0.2 | 0.81 ± 0.12 | 14.1 ± 0.3 | 25.1 ± 1.2 |
| Model | 66.5 ± 5.0## | 55.0 ± 3.5## | 8.3 ± 1.1## | 1.12± 0.15# | 18.8 ± 0.3## | 15.3 ± 1.3## |
| sh-NC | 70.5 ± 3.7 | 68.0 ± 2.0 | 8.0 ± 1.7 | 1.33± 0.2 | 19.6 ± 0.11 | 14.3 ± 0.6 |
| sh-AHNAK | 89.1 ± 3.6** | 88.3 ± 4.8** | 5.9 ± 0.4** | 0.90 ± 0.11* | 15.4 ± 0.3** | 18.2 ± 2.0** |
| Colchicine | 80.5 ± 2.2** | 74.0 ± 4.8* | 6.2 ± 0.23** | 0.92 ± 0.3* | 17.4 ± 0.24* | 21.7 ± 1.9** |
| R4F-NM@F127-Col | 100.2 ± 2.0** | 85.0 ± 2.1*s* | 4.4 ± 0.3** | 0.78 ± 0.27** | 14.2 ± 0.28** | 26.0 ± 1.5** |

Note: Values are means ± SEM (n = 5). # p < 0.05 and ## p < 0.01 as compared with control group; * p < 0.05 and ** p < 0.01 as compared with Model group.
