# Supplementary figures and images for: Harnessing Nanotechnology for Gout Therapy: Colchicine-Loaded Nanoparticles Regulate Macrophage Polarization and Reduce Inflammation
Source: Biomater Res. 2024 Dec 11;28:0089. doi: 10.34133/bmr.0089 (PMC11632155; doi:10.34133/bmr.0089)

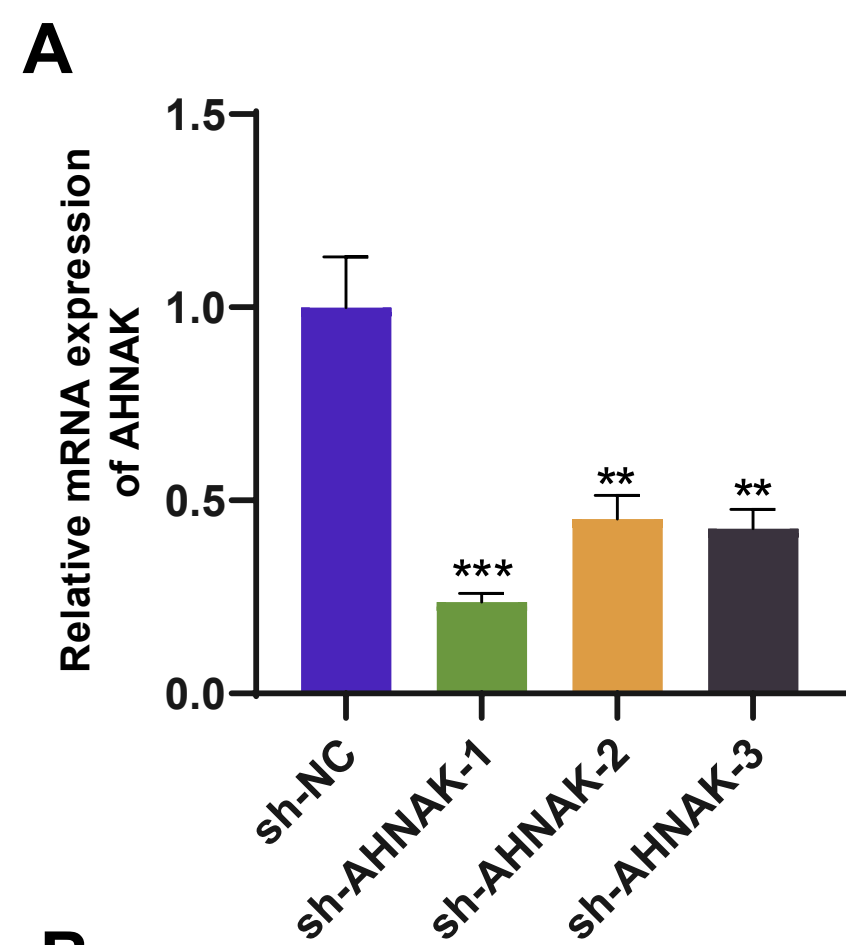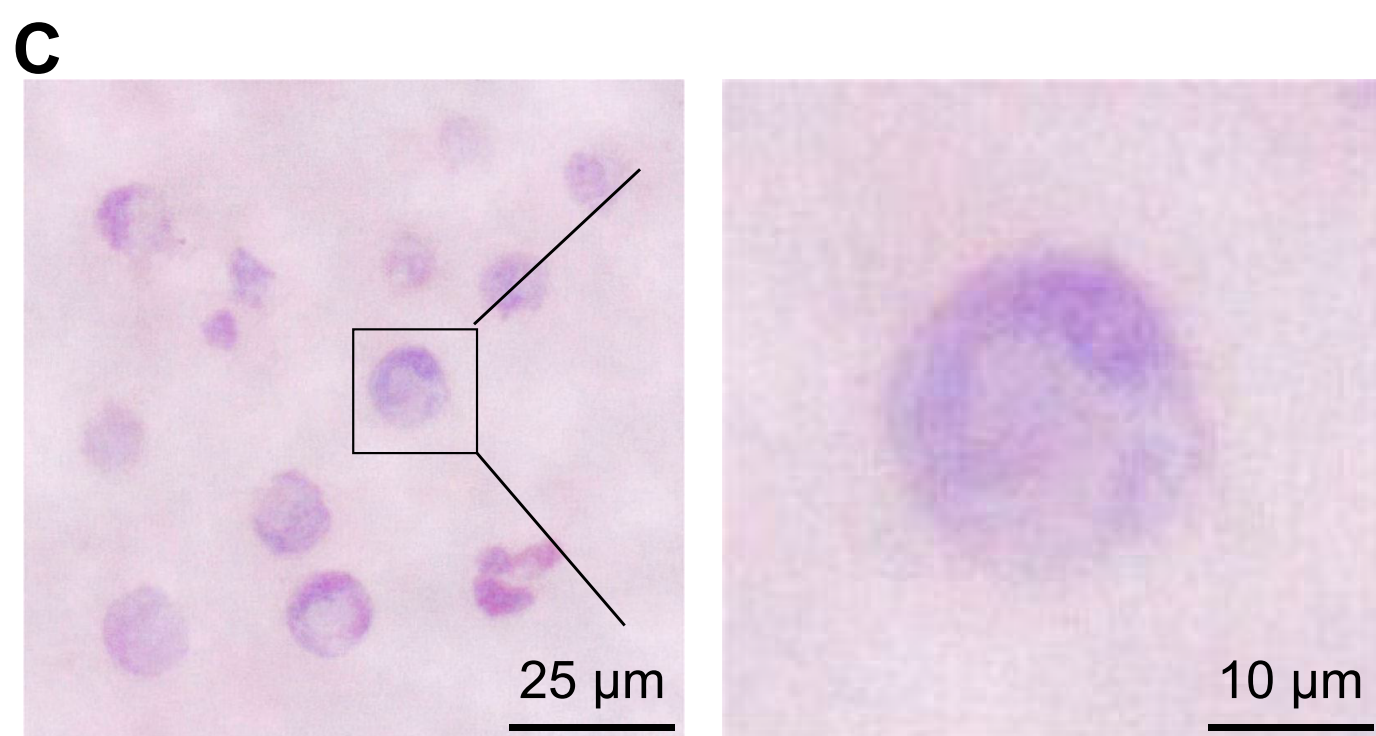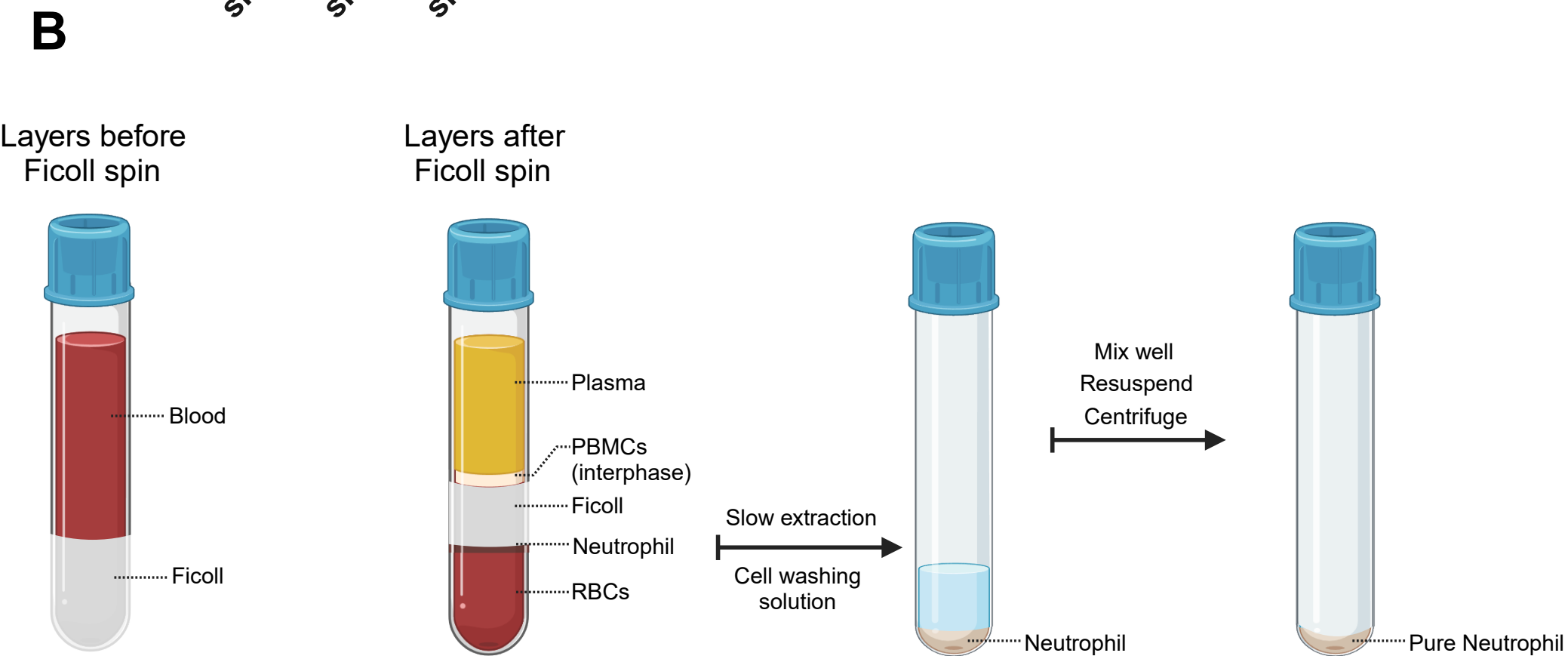

Supplement: Supplementary 1 — Figs. S1 to S8 Tables S1 to S7 [file bmr.0089.f1.zip › Figure S1.pdf]

**A**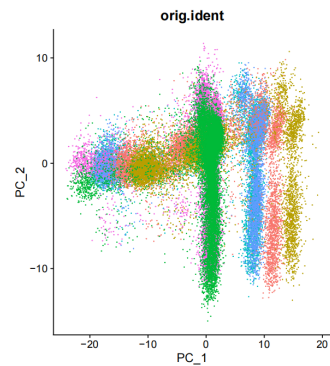**B**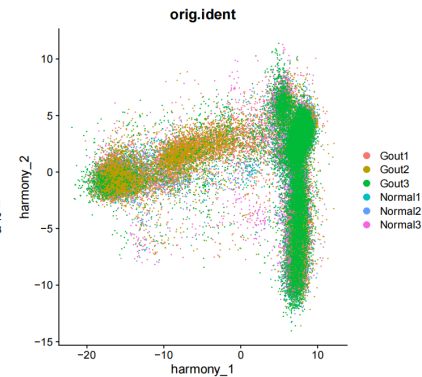**C**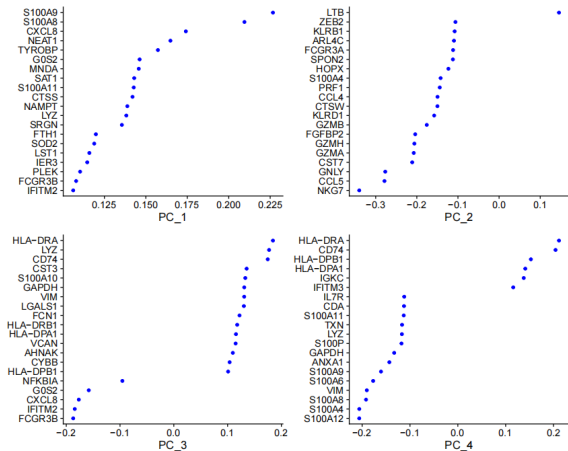**D**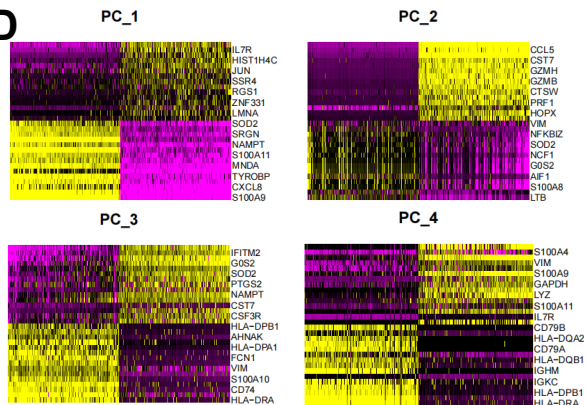**E**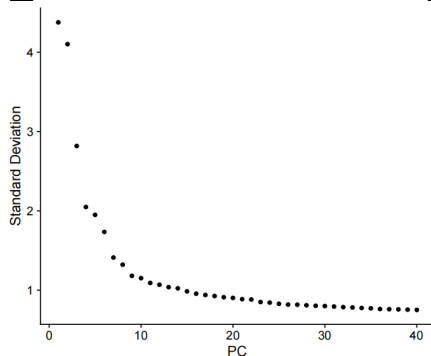**F**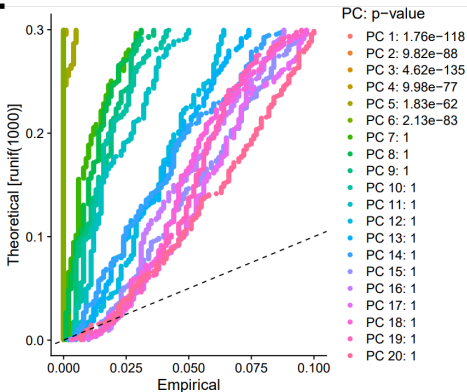

Supplement: Supplementary 1 — Figs. S1 to S8 Tables S1 to S7 [file bmr.0089.f1.zip › Figure S3.pdf]

**A**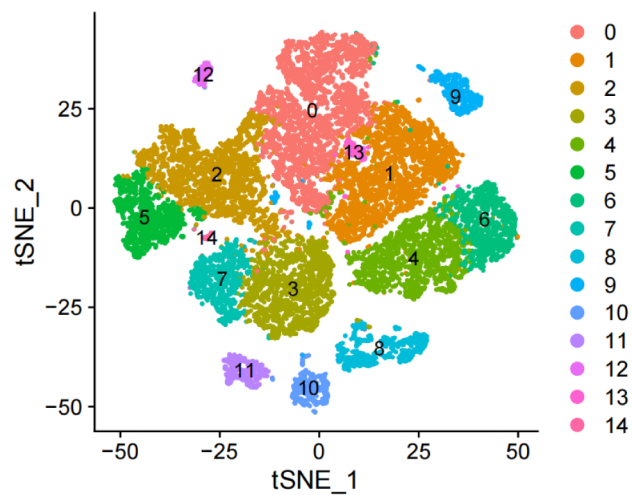**B**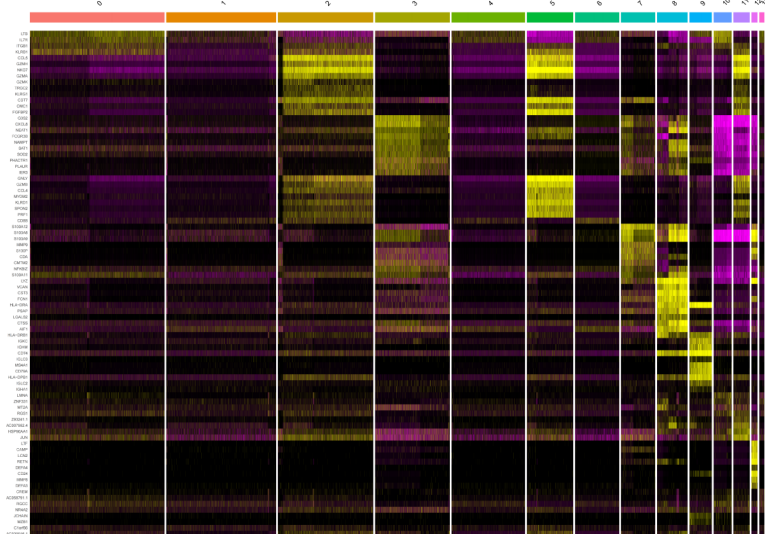**C**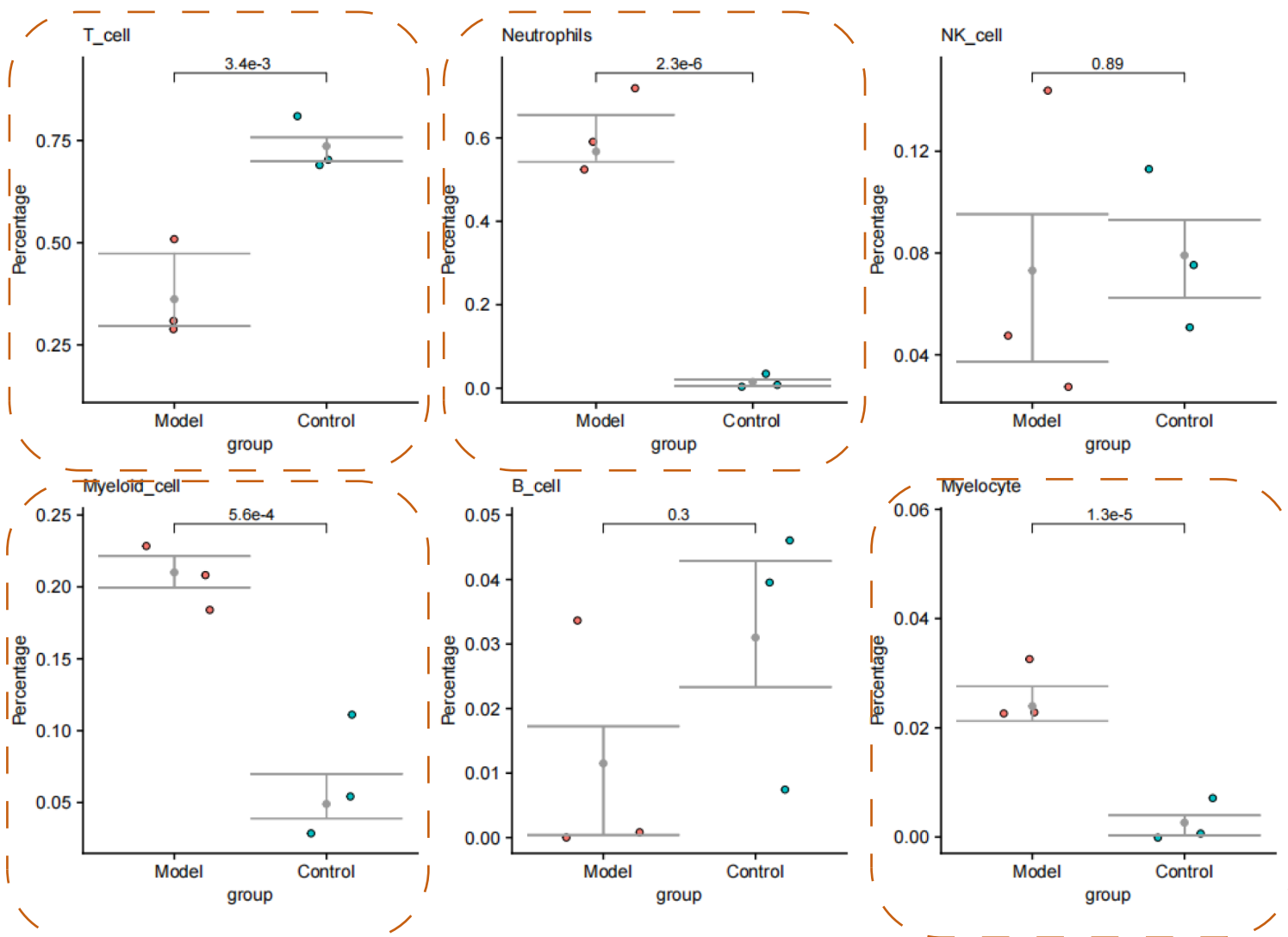

Supplement: Supplementary 1 — Figs. S1 to S8 Tables S1 to S7 [file bmr.0089.f1.zip › Figure S4.pdf]

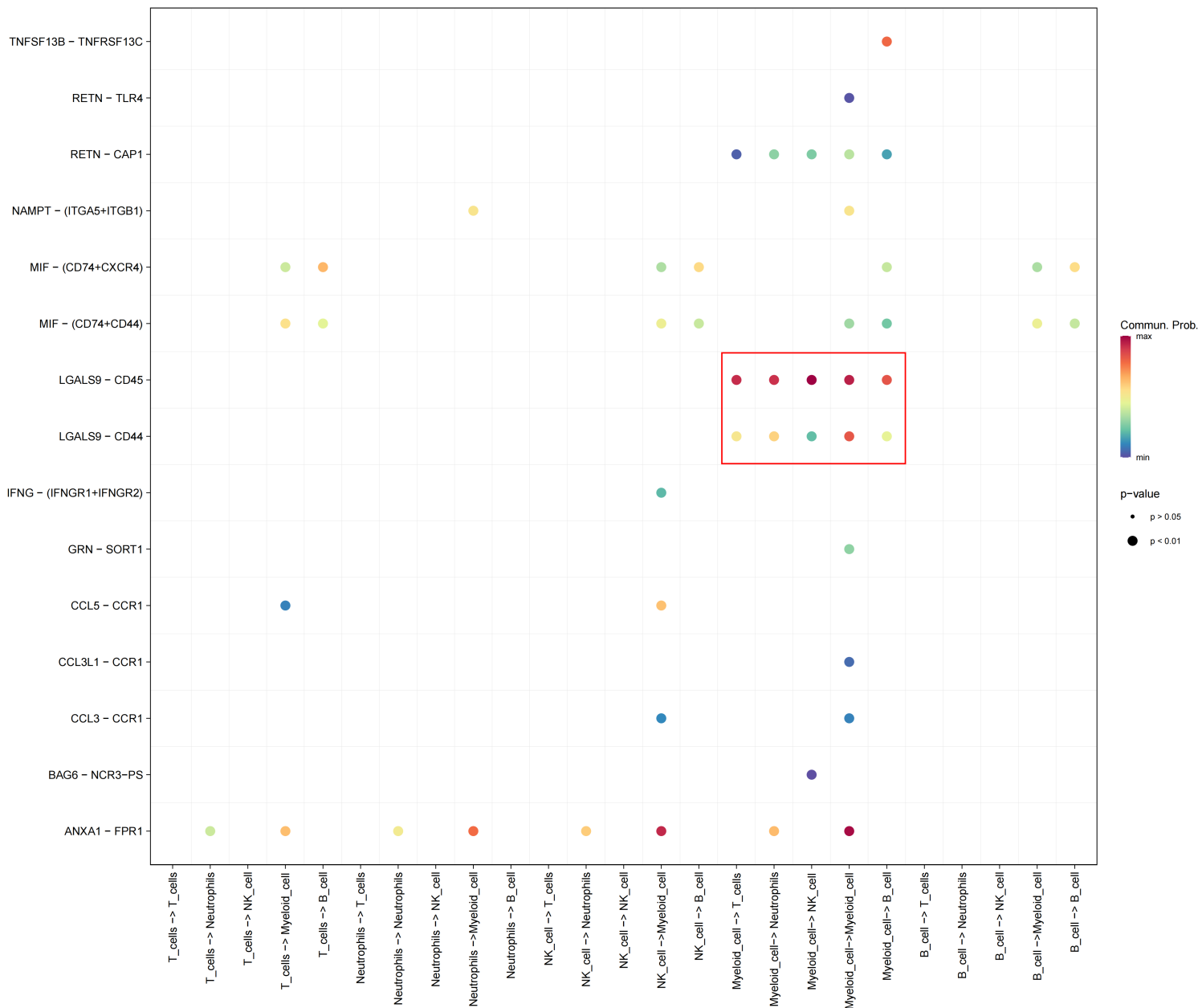

Supplement: Supplementary 1 — Figs. S1 to S8 Tables S1 to S7 [file bmr.0089.f1.zip › Figure S5.pdf]

**A**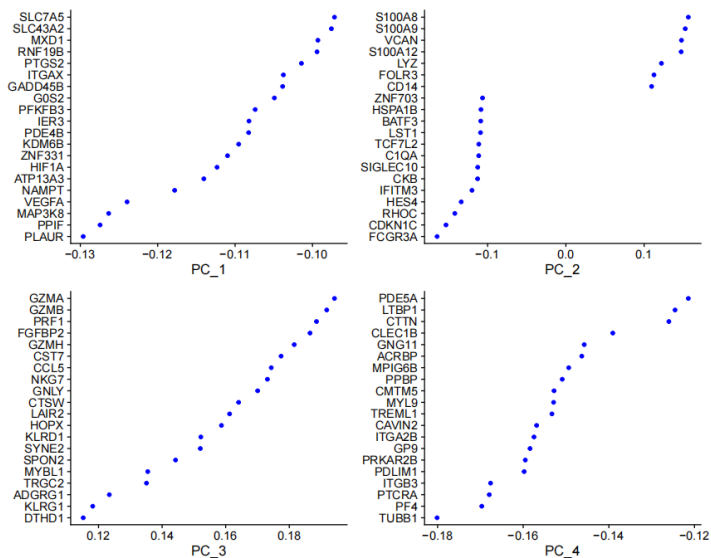**B**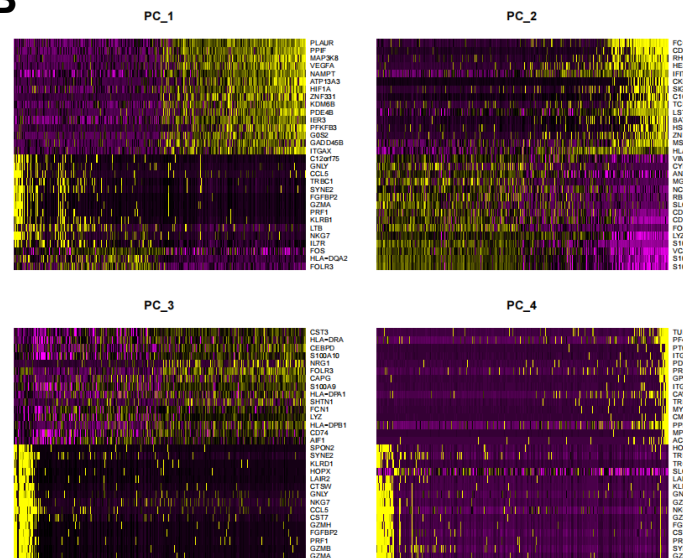**C**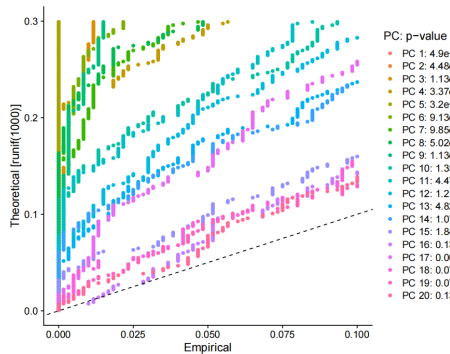**D**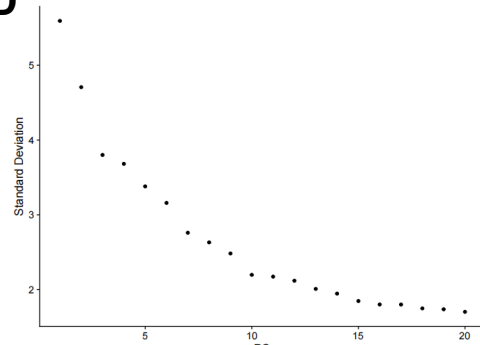**E**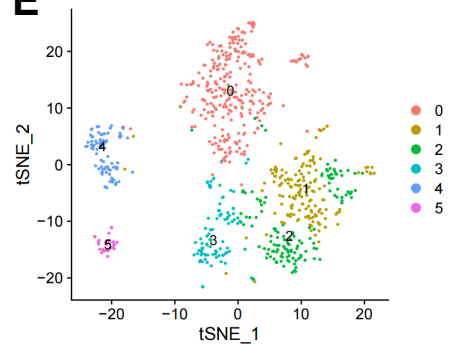**F**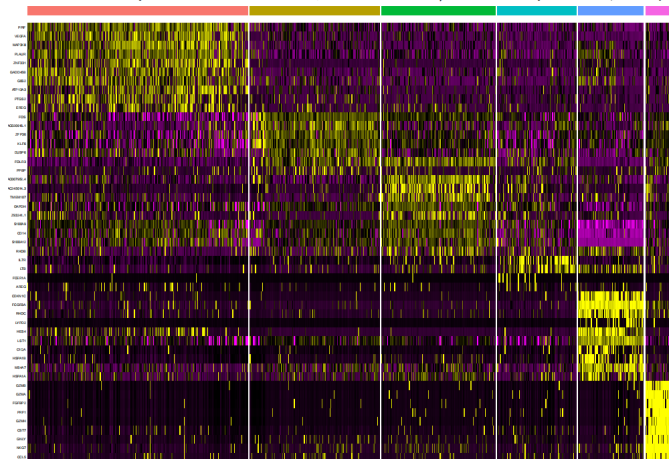**G**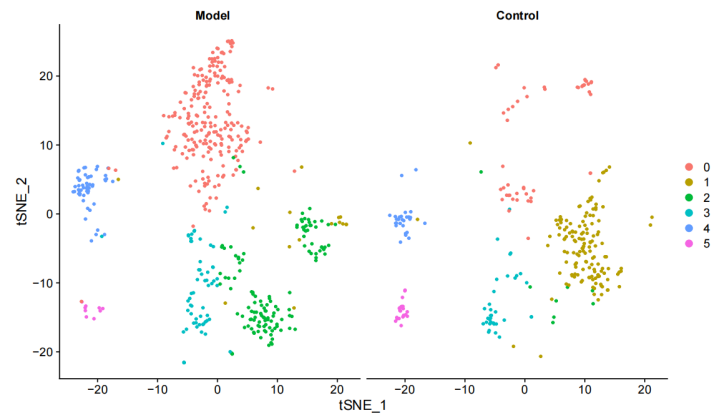

Supplement: Supplementary 1 — Figs. S1 to S8 Tables S1 to S7 [file bmr.0089.f1.zip › Figure S6.pdf]

**A****Size Distribution by Intensity**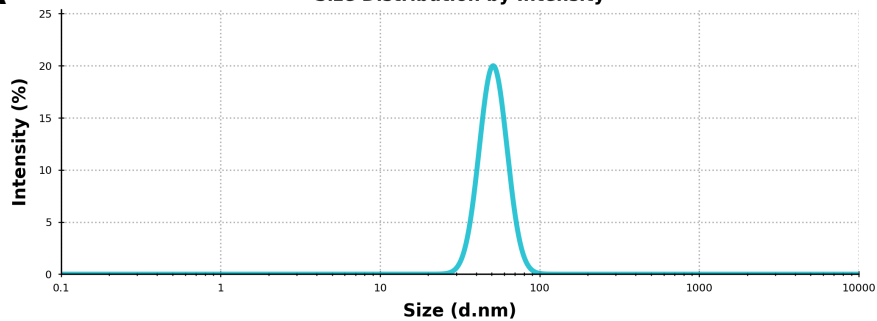**B**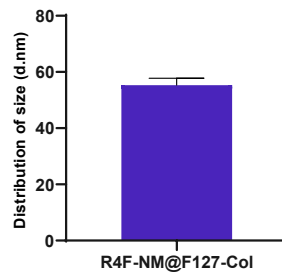**C**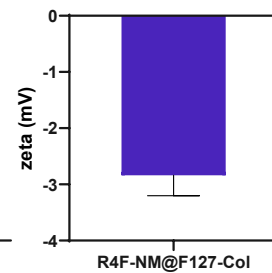**D**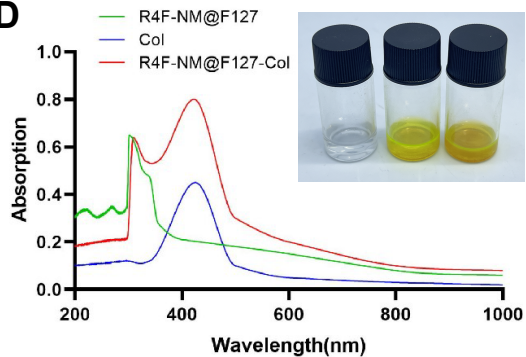**F**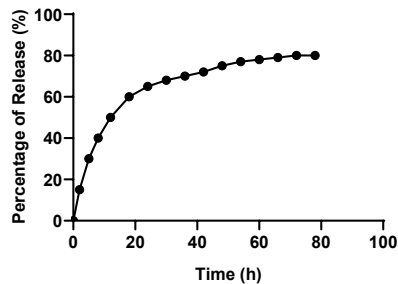**G**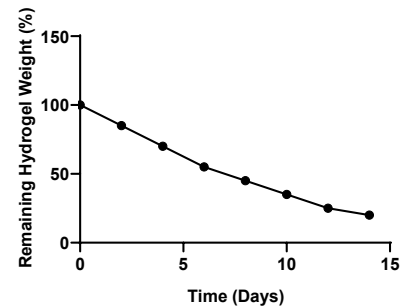**E**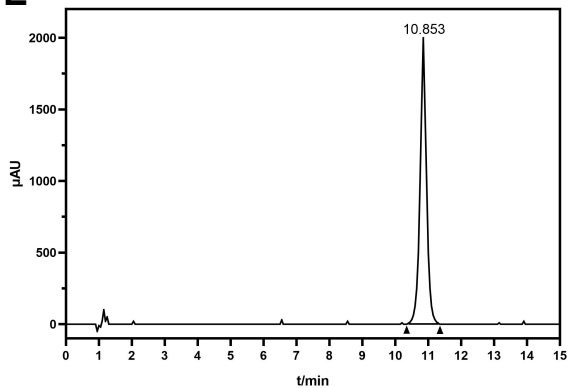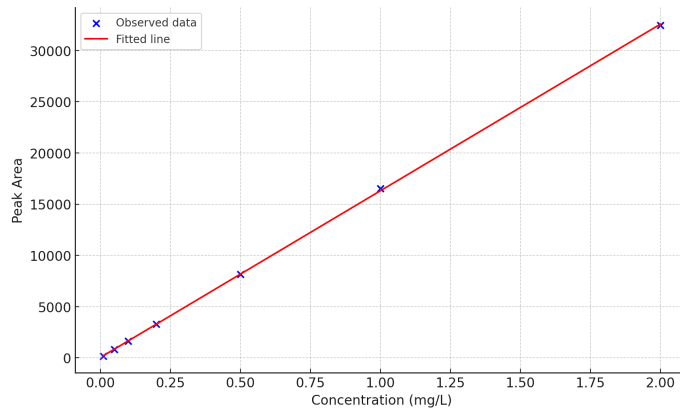

Supplement: Supplementary 1 — Figs. S1 to S8 Tables S1 to S7 [file bmr.0089.f1.zip › Figure S7.pdf]

R4F-NM@F127

Heart

Liver

## Spleen

Lung

## Kidney

## Brain

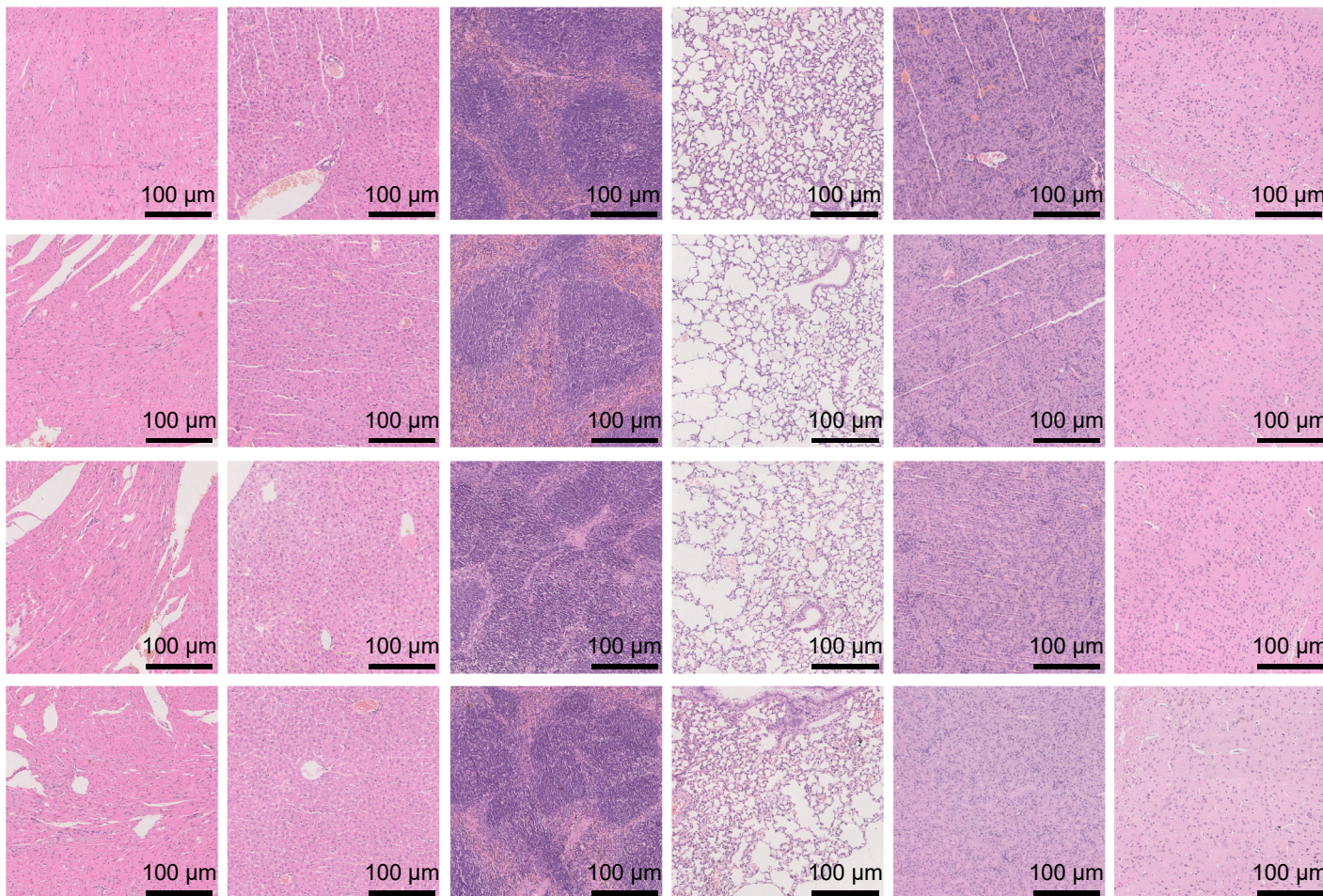

# B

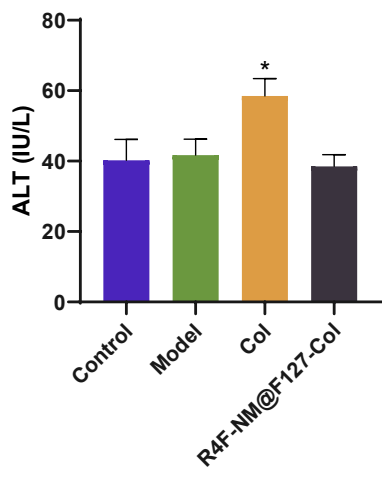

**C**

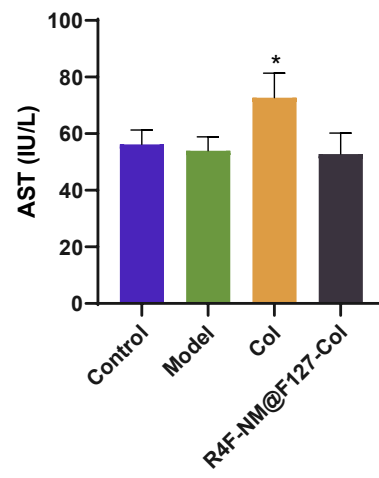

Supplement: Supplementary 1 — Figs. S1 to S8 Tables S1 to S7 [file bmr.0089.f1.zip › Figure S8.pdf]
